# Exploring neuronal mechanisms involved in the scratching behavior of a mouse model of allergic contact dermatitis by transcriptomics

**DOI:** 10.1186/s11658-022-00316-w

**Published:** 2022-02-19

**Authors:** Boyu Liu, Ruixiang Chen, Jie Wang, Yuanyuan Li, Chengyu Yin, Yan Tai, Huimin Nie, Danyi Zeng, Junfan Fang, Junying Du, Yi Liang, Xiaomei Shao, Jianqiao Fang, Boyi Liu

**Affiliations:** 1 Department of Neurobiology and Acupuncture Research, The Third Clinical Medical College, Key Laboratory of Acupuncture and Neurology of Zhejiang Province, Zhejiang Chinese Medical University, Hangzhou, 310053 China; 2 The First Department of Acupuncture, Shaanxi Hospital of Traditional Chinese Medicine, Xi’an, Shaanxi China; 3 Academy of Chinese Medical Sciences, Zhejiang Chinese Medical University, Hangzhou, China

**Keywords:** Itch, Pain, Sensory neurons, Allergic contact dermatitis, RNA-seq

## Abstract

**Background:**

Allergic contact dermatitis (ACD) is a common skin condition characterized by contact hypersensitivity to allergens, accompanied with skin inflammation and a mixed itch and pain sensation. The itch and pain dramatically affects patients’ quality of life. However, still little is known about the mechanisms triggering pain and itch sensations in ACD.

**Methods:**

We established a mouse model of ACD by sensitization and repetitive challenge with the hapten oxazolone. Skin pathological analysis, transcriptome RNA sequencing (RNA-seq), qPCR, Ca^2+^ imaging, immunostaining, and behavioral assay were used for identifying gene expression changes in dorsal root ganglion innervating the inflamed skin of ACD model mice and for further functional validations.

**Results:**

The model mice developed typical ACD symptoms, including skin dryness, erythema, excoriation, edema, epidermal hyperplasia, inflammatory cell infiltration, and scratching behavior, accompanied with development of eczematous lesions. Transcriptome RNA-seq revealed a number of differentially expressed genes (DEGs), including 1436-DEG mRNAs and 374-DEG-long noncoding RNAs (lncRNAs). We identified a number of DEGs specifically related to sensory neuron signal transduction, pain, itch, and neuroinflammation. Comparison of our dataset with another published dataset of atopic dermatitis mouse model identified a core set of genes in peripheral sensory neurons that are exclusively affected by local skin inflammation. We further found that the expression of the pain and itch receptor MrgprD was functionally upregulated in dorsal root ganglia (DRG) neurons innervating the inflamed skin of ACD model mice. MrgprD activation induced by its agonist β-alanine resulted in exaggerated scratching responses in ACD model mice compared with naïve mice.

**Conclusions:**

We identified the molecular changes and cellular pathways in peripheral sensory ganglia during ACD that might participate in neurogenic inflammation, pain, and itch. We further revealed that the pain and itch receptor MrgprD is functionally upregulated in DRG neurons, which might contribute to peripheral pain and itch sensitization during ACD. Thus, targeting MrgprD may be an effective method for alleviating itch and pain in ACD.

**Supplementary Information:**

The online version contains supplementary material available at 10.1186/s11658-022-00316-w.

## Background

Allergic contact dermatitis is a type of skin disease involving overreactivity of skin to contact allergens, accompanied with skin inflammation and mixed pain and itch sensations [1–4]. ACD is triggered by sensitization and subsequent contact with allergens, which may originate from occupational, environmental, or nutritional sources [5]. Itch and pain are typical complaints that pose heavy healthy burdens, both physically and mentally, to the patients [6, 7]. The repetitive pain and itch sensation usually triggers constant scratching towards the affected skin, which may disrupt the skin barrier and promotes invasion of allergens and pathogens and further exacerbates skin inflammation [8].

The immune responses and mechanisms underlying ACD have been relatively well studied [9]. However, still little is known about the mechanisms triggering pain and itch in ACD. The pain and itch sensation is triggered by the excitation of the free nerve endings of sensory nerves that innervate the skin [10]. These free nerve endings can be directly activated by a variety of pain- and itch-inducing substances [10–13]. Evidence suggests that immune/skin cell–neuron crosstalk can drive and promote chronic pain and itch [10]. For example, our recent study suggests that IL-33, which is produced by keratinocytes upon ACD, can act upon neuronal receptor ST2 to trigger sensory neuron hyperexcitability and induce scratching behavior [14]. In addition, CXCL10, which is released by neutrophils under conditions of ACD and atopic dermatitis, acts upon neuronal CXCR3 receptor to promote itch via activation of sensory neurons [15, 16]. The inflammatory mediators released from immune or skin cells upon inflammation may also sensitize the sensory nerve and further exacerbate pain and itch sensation [17].

The dorsal root ganglion (DRG) is enriched with primary sensory neurons that receive sensory information from peripheral nerve endings and transfer the signals to spinal cord. It is critically involved in sensory signal integration, transduction, and sensitization [10, 18, 19]. It is known that peripheral inflammation can induce the activation of diverse signaling pathway and inflammatory responses in peripheral tissues and DRG. However, how ACD-induced local skin inflammation affects the innervating peripheral sensory ganglia remains largely unknown.

In this study, we combined the established mouse ACD model and skin pathology and behavior assay with RNA-seq to explore transcriptome changes in DRG innervating the inflamed skin. Based upon transcriptome technique, we were able to identify the molecular changes and cellular signaling pathways in DRG during ACD. We further analyzed the expression of long noncoding RNAs (lncRNAs) and their potential interaction with mRNAs in DRG in the context of ACD. Bioinformatics analysis, combined with qPCR/protein validation and functional assay, revealed that the pain and itch receptor MrgprD is functionally upregulated in DRG neurons, which may contribute to peripheral pain and itch sensitization during ACD.

## Material and methods

### Experimental reagents

Oxazolone was purchased from Alfa Aesar (Thermo Fisher Scientific, USA). Beta-alanine was purchased from Sigma (Merck, USA). Olive oil was purchased from Sigma (Merck, USA).

### Animals

C57BL/6 mice (6–8 weeks of age, male) were used in the present study. Animals were housed in Zhejiang Chinese Medical University Laboratory Animal Facility (five animals per cage, 12 h dark–light cycle, 24 ± 2 ℃). The cages were standard breeding cages for mice (325 mm × 210 mm × 180 mm). Animals were given free access to water and food. The food was standard mouse chow (protein 18%, fat 5%, and fiber 5%). All animals were allowed > 7 days to accommodate the breeding facility ahead of any assay or test.

### Oxazolone-induced mouse ACD model

The model was established as we described previously [12]. Briefly, the mice were first sensitized by applying 30 μl 2.0% (wt/vol) oxazolone (a mixture of olive oil in acetone at a ratio of 1:4) to the shaved belly. The hair on the belly and nape of the neck was removed by an electronic shaver. To reduce the stress of the mice during the shaving process, the mice were anesthetized by isoflurane inhalation. Five days (at day 0) later, a lower concentration of oxazolone (at a concentration of 0.5% dissolved in acetone, in a volume of 40 μl) was painted on the shaved nape of the neck of the sensitized mice by pipetting. Mice were challenged with oxazolone every other day for a total of ten challenges.

### Scratching behavioral analysis

Behavioral experiments were performed on days 5, 11, and 17. Animals were put in the chamber for behavioral observation. They were accommodated to the testing environment for 45 min before recording. The mouse scratching movements to the nape of the neck were recorded with video cameras that were placed underneath the behavioral observation chamber. One scratching bout was defined as a single or a series of scratching actions of the hindpaws to the neck area that ended with either the animals putting the hindpaws back on the floor mesh or licking the hindpaws. All behavioral tests were performed by an experimenter blinded to experimental conditions.

### Skin bi-fold thickness measurement

Bi-fold skin thickness was measured via a digital caliper as we described previously [13]. The bi-fold skin thickness was measured before sensitization and during the challenging phase. Six measurements were obtained from different sites in the dorsal part of the skin of each animal. The values were then averaged and summed.

### Skin histopathological assessment

The dermatitis score was determined at day 18, 2 h after the last (tenth) oxazolone challenge, in living animals from three perspectives, namely, excoriation, scarring, and erythema, using four evaluation scales (values 0–3 for none, mild, moderate, and severe, respectively) by an experimenter blinded to the groupings. The dermatitis score was judged by criteria described previously [12]. After evaluation, the mice were euthanized by overdose isoflurane inhalation. Neck skin was harvested from mice and fixed with 10% paraformaldehyde. Hematoxylin- and eosin-stained skin paraffin sections were used for morphological assessment using a microscope with transmitted light (Leica, Germany). Epidermal thickness and the number of CD3^+^ cells infiltrated in the skin were calculated by an experimenter blinded to the groupings.

### Tissue collection and RNA extraction

The mice were euthanized by overdose isoflurane inhalation. Bilateral C1–T1 DRGs were dissociated immediately after skin tissue collection as mentioned above under a microscope and then dropped into RNAlater stabilization solution (Thermo Fisher Scientific). Total RNA was extracted by means of TRIzol reagent (Thermo Fisher Scientific, USA). DNase I was added to eliminate DNA contamination. Subsequently, total RNA was qualified and quantified using 2100 Bioanalyzer System (Agilent, USA) and NanoDrop 2000 Spectrophotometer (Thermo Fisher Scientific, USA) as described [20].

### Long noncoding RNA library construction

The procedures have been described in our previous study [21]. Briefly, Ribo-Zero Magnetic Kit was used for processing total RNA (1 μg per sample) to eliminate rRNAs. Then the RNAs were further fragmented via addition of First-Strand Master Mix (Invitrogen). First-strand cDNAs were obtained via reverse transcription using random primers. A second-strand cDNA synthesis was followed thereafter. The cDNA fragments were then enriched by many cycles of PCR amplification. After that, PCR clean-up was carried out using AMPure XP (Beckman Coulter). The established PCR libraries were then qualified using qPCR and Agilent 2100 Bioanalyzer System. Hiseq 4000 platform was used for the final sequencing of the RNA libraries by BGI of Shenzhen, China.  The procedures of bioinformatics analysis and data deposition have been described in Additional file 1. The transcriptome RNA-seq containing both mRNA and lncRNA datasets is provided as Additional file 2: Table S1 and Additional file 3: Table S2.

### Cluster analysis and screening of DEGs

The analysis of gene expression was carried out with the “DESeq2” package of R software using Bioconductor. The “Pheatmap” package of R software (version 3.5.1; URL http://www.R-project.org) was utilized to analyze gene expression cluster as well as patterns. The screening and illustrations of DEGs (including mRNA and lncRNA) were obtained via scatter plot as reported previously [22]. The criteria of fold change ≥ 1.25 and *q* value ≤ 0.001 were established to screen differentially expressed mRNA and lncRNA genes.

### DEGs enrichment analysis

Dr. Tom system (BGI, Shenzhen, China) was used to perform functional enrichment analysis of DEGs. Bubble plots of KEGG and GO enrichment were drawn by “ggplot” package in R software.

### qPCR testing

Total RNA extracted from DRG tissue was reverse transcribed into cDNAs with PrimeScript RT Reagent Kit (Takara Bio Inc., Japan). Detailed information regarding the primer sequence is provided in Additional file 4: Table S3. qPCR was conducted by LightCycler480 System (Roche, Switzerland). TB Green Premix Ex Taq II (Takara Bio Inc, Japan) was used as the master kit. Beta-actin was used as the housekeeping gene. All reactions were performed in triplicate. The cycle threshold (CT) value was deduced from the analyzing software provided by LightCycler480 System. The △△Ct method was utilized to calculate gene expression fold changes [23, 24].

### Immunofluorescence and immunohistochemistry staining

The detailed methods regarding tissue processing, embedding, sectioning, and antibody incubation are described in our previous publications [25, 26]. We used the following antibodies: rabbit anti-CD3 (1:200, no. MA1-90582, Thermo Fisher, USA), rabbit anti-MrgprD (1:200, no. AMR-061, Alomone, Israel), and mouse anti-NeuN (1:300, no. ab104224, Abcam, UK). Secondary antibodies were as follows: donkey anti-rabbit IgG H&L (Alexa Fluor 488, 1:500, no. ab150065, Abcam, UK) and donkey anti-mouse IgG H&L (Alexa Fluor 647, 1:500, no. ab150111, Abcam, UK). For immunohistochemistry, the expression of CD3 was visualized using ABC kits (Vector Laboratories, USA) according to the manufacturer’s instructions. The immunostaining pictures were recorded with a laser scanning confocal microscope (Nikon, Japan). Uniform microscope settings were maintained throughout all image capture sessions. The images were analyzed by an observer blinded to groupings. Four to five images were randomly selected per sample and then analyzed, averaged, and compared.

### Drug application

For naïve mice, β-alanine [10, 30, and 100 mM dissolved in phosphate-buffered saline (PBS)] was subcutaneously (50 μl injection volume) injected into the shaved neck skin under constraint. For oxazolone-induced ACD mice, β-alanine (30 mM) was subcutaneously (50 μl injection volume) injected into the shaved neck skin under constraint 1 day after the last oxazolone (the tenth) challenge. Vehicle group mice received only PBS injection (50 μl injection volume). Scratching behavioral test was performed right after β-alanine/PBS injection.

### Cell culture and Ca^2+^ imaging

Bilateral C1–T1 DRGs were harvested and digested using dispase and collagenase type 1 (Gibco, USA) described in our previous study [27]. DRG neurons were cultured in Dulbecco’s Modified Eagle Medium (DMEM) plus 10% fetal bovine serum (FBS) (Hyclone, USA) on round coverslips coated with poly-d-lysine (Sigma, USA). Cells were incubated with Fura-2AM (10 μM concentration, Abcam, UK, dissolved in DMEM) for 45 min. Cells were subsequently washed three times and imaged in the loading buffer (containing 140 NaCl, 5 KCl, 2 CaCl_2_, 2 MgCl_2_, and 10 HEPES, pH 7.4 with NaOH).

Ca^2+^ imaging was carried out with the Nikon ECLIPSE Ti-S (Nikon, Japan) microscope. Polychrome V monochromator (Till Photonics, USA) was used as the light source. Orca Flash 4.0 CCD camera (Hamamatsu, Japan) was used for capturing images. MetaFluor software (Molecular Devices, USA) was used for image data processing. The pseudo-color Ca^2+^ images were produced by ImageJ software. We considered a cell as positively responding if its Ca^2+^ spike jumped over 20% to its original baseline, as reported previously [28, 29].

### Statistical analysis

Comparisons between two groups were made using Student’s *t*-test, whereas three-group comparisons were made using one-way or repeated two-way analysis of variance (ANOVA). ANOVA was then followed by Tukey’s post hoc test. Differences were considered statistically significant at *p* values less than 0.05. Data are presented as the mean ± standard error of the mean (SEM).

## Results

### Oxazolone-induced ACD model mice showed remarkable skin inflammation and persistent scratching behavior

**Fig. 1:**
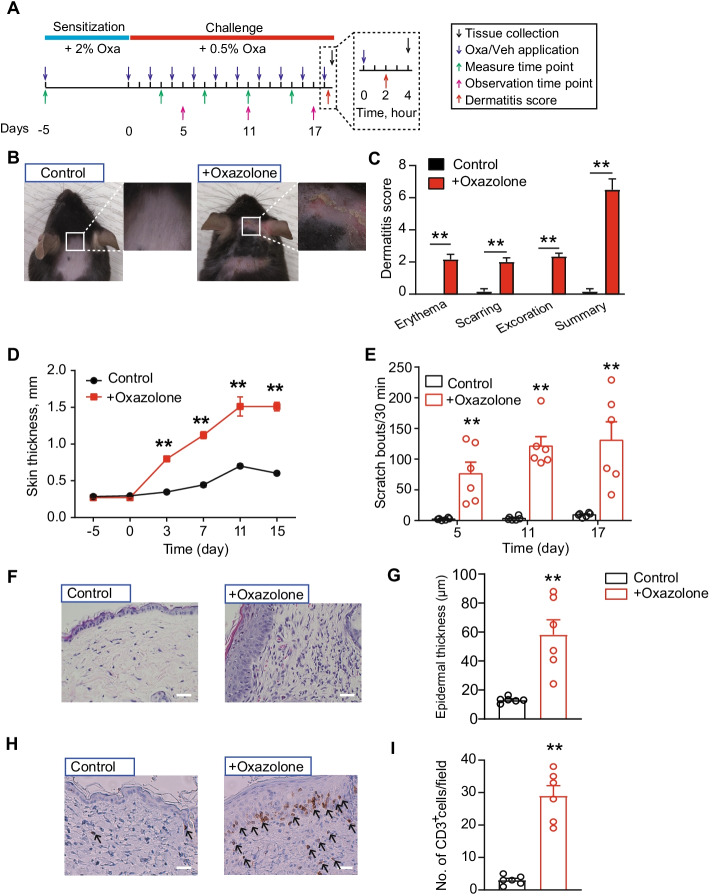
The establishment of the mouse model of ACD by oxazolone sensitization and challenge and evaluations. **A** Protocol for the establishment of the oxazolone (Oxa)-induced ACD model in mice. Control group receive vehicle (acetone) treatment only. **B** Typical pictures showing the neck skin condition of mice in vehicle- or oxazolone-treated group. **C** Pathological dermatitis scores of control and oxazolone group mice. **D** Bi-fold skin thickness evaluation of the neck skin of control and oxazolone group of mice. **E** Scratching behavior of mice treated with oxazolone or vehicle. **F** Representative pictures of hematoxylin and eosin (H&E) staining of neck skin of control and oxazolone group of mice. **G** Summary of epidermal thickness of control and oxazolone group mice. **H** Representative pictures of immunohistochemistry staining of CD3^+^ cells in neck skin of control and oxazolone group mice. **I** Summary of the number of CD3^+^ cells per observation field. *n* = 6 mice per group. **p < 0.01 versus control group. Scale bar, 100 μm

Previous studies from our group and others have shown that initial sensitization and repeated challenges with the hapten oxazolone triggers ACD-like symptoms in mice [12, 13, 30]. These symptoms include skin dryness, erythema, excoriation, edema, inflammatory cell infiltration, and itch- and pain-like behavior, accompanied with the development of eczematous lesions. We then established the mouse model of ACD according to protocols established as mentioned above. As shown in Fig. 1A, mice were initially sensitized by applying oxazolone (2%) to the abdominal skin, followed by ten oxazolone (0.5%) challenges on neck skin. The control group of mice received only vehicle (acetone) challenges. After a total of ten challenges, oxazolone-challenged mice developed obvious skin erythema, excoriation, dryness, and edema (Fig. 1B–D). Oxazolone-challenged mice developed robust scratching behavior toward the neck skin over the challenging period (Fig. 1E). Skin pathological analysis further indicated obvious epidermal hyperplasia (Fig. 1F, G), accompanied with CD3^+^ cell infiltration in both epidermis and dermis (Fig. 1H, I). These signs are consistent with previous studies [12], demonstrating the successful establishment of the ACD mouse model.

### Gene expression profiling in DRG of ACD model mice by transcriptomics

**Fig. 2:**
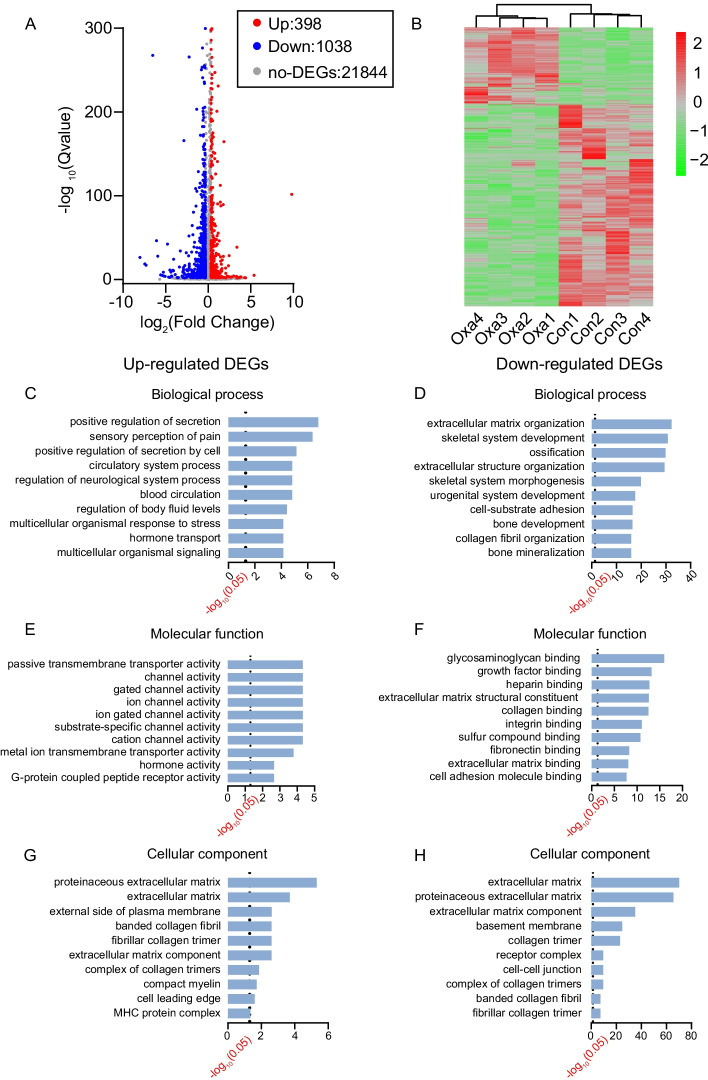
Bioinformatics analysis of DRG from oxazolone-induced mouse ACD model. **A** Volcano graph of mRNA expression in DRG of oxazolone (Oxa) group versus control (Con) group. Dots with different colors indicate corresponding expression changes. **B** Heatmap illustration of the hierarchical clustering of DEmRNAs of oxazolone group versus control group. **C**, **E**, and **G** The top ten most significantly enriched biological processes, molecular functions, and cellular components of upregulated DEmRNAs. **D**, **F**, and **H** The top ten most significantly enriched biological processes, molecular functions, and cellular components of downregulated DEmRNAs. The dotted line indicates *q*-value of 0.05

To identify the genes possibly involved in mediating the scratching response of ACD model mice, we harvested bilateral C1–T1 DRG innervating the neck skin from both oxazolone-challenged and control group of mice for RNA-seq. We managed to obtain RNA of high quality that meets the criterion for RNA-seq (Additional file 5: Fig. S1). Basic information regarding the quality of the RNA-seq dataset is presented in Table 1. RNA-seq identified a total of 23,280 mRNAs and 25,007 lncRNAs. The criteria of fold change ≥ 1.25 and *q* value ≤ 0.01 were established to screen differentially expressed mRNA and lncRNAs genes (DEmRNAs and DElncRNAs, respectively). Based on the criteria, we managed to identify a total of 1436 DEmRNAs (including 398 up- and 1038 downregulated) and 374 DElncRNAs (including 77 up- and 297 downregulated). These DEmRNAs and DElncRNAs were displayed in volcano plot as in Fig. 2A and Additional file 6: Fig. S2A and further summarized in heatmap as in Fig. 2B and Additional file 6: Fig. S2B. Hierarchical clustering analysis demonstrated a clear segregation between the oxazolone-challenged group and the control group, but no segregation within groups (Fig. 2B and Additional file 6: Fig. S2B).

**Table 1:** Total reads and mapping ratio for control and model groups in RNA-seq

Sample	Total raw reads (Mb)	Total clean reads (Mb)	Clean reads Q30 (%)	Clean reads ratio (%)	Total mapping ratio (%)
Control 1	121.49	114.27	95.85	94.06	97.10
Control 2	121.31	113.86	95.86	93.86	97.33
Control 3	121.32	113.89	95.70	93.87	97.31
Control 4	121.53	114.23	95.53	93.99	96.86
Model 1	120.90	113.43	95.39	93.82	96.82
Model 2	121.41	113.76	95.77	93.70	97.13
Model 3	121.41	113.96	96.07	93.87	97.29
Model 4	121.36	113.93	95.74	93.87	97.28

### Examination of differentially expressed genes in DRG tissues of ACD mice

Among all of the DEmRNAs we have identified, we were able to retrieve a number of genes well known to be involved in pain or itch signal processing. These genes include *Cckbr* (cholecystokinin B receptor, fold change 5.2), *Vgf* (nerve growth factor inducible, fold change 2.8), *Bdnf* (brain-derived neurotrophic factor, fold change 2.23), *Atf3* (activating transcription factor 3, fold change 1.97), *Il31ra* (interleukin 31 receptor A, fold change 1.7), and *Nppb* (natriuretic peptide receptor 1, fold change 1.43). In addition, 28 DEmRNAs had expression that was altered by tenfold or more, including 6 upregulated and 46 downregulated. These genes include *Gdf1* (growth differentiation factor 1, fold change 891.4), *Sprr1a* (small proline-rich protein 1A, fold change 10.3), and *Tmem184a* (transmembrane protein 184a, fold change 10.1). Twenty-five genes showed expression changes between five and tenfold, including 5 upregulated and 20 downregulated. For DElncRNAs, 12 DElncRNAs showed expression that was altered by more than tenfold, including seven upregulated and five downregulated. These DElncRNAs included *Gm39752* (fold change 64.4), *Gm30259* (fold change 64.1), and *Gm7334* (fold change 60.97). Seven genes showed expression changes between five and tenfold, with three upregulated and four downregulated. Detailed information regarding the top 20 up- or downregulated DEmRNAs and DElncRNAs is presented in Tables 2, 3, 4, 5.

**Table 2:** Detailed information on the top 20 upregulated DEmRNAs

Upregulated mRNA	Gene ID	Location	*Q* value	Log_2_ fold-change (Oxa/Veh)	Official gene name (NCBI)
*Gdf1*	14559	Chr8: 70768425–70784242	2.04 × 10^−102^	9.828536645	Growth differentiation factor 1
*4933402N22Rik*	545732	Chr5: 11968010–11972771	1.15 × 10^−5^	5.383011508	RIKEN cDNA 4933402N22 gene
*LOC102641086*	102641086	Chr17: 328351–328761	6.79 × 10^−4^	3.850459002	–
*LOC100862456*	100862456	Chr17: 328238–328649	6.79 × 10^−4^	3.850459002	–
*Sprr1a*	20753	Chr3: 92391261–92393188	2.56 × 10^−39^	3.371649014	Small proline-rich protein 1A
*Tmem184a*	231832	Chr5: 139790707–139805725	1.78 × 10^−4^	3.337701682	Transmembrane protein 184a
*Krt14*	16664	Chr11: 100093988–100098336	8.80 × 10^−5^	3.004277949	Keratin 14
*Fermt1*	241639	Chr2: 132746097–132787956	7.35 × 10^−4^	2.752739182	Fermitin family member 1
*Ecel1*	13,599	Chr1: 87070596–87084807	3.96 × 10^−29^	2.459007979	Endothelin-converting enzyme-like 1
*BGIG10090_45383*	BGIG10090_45383	–	6.26 × 10^−4^	2.436576933	–
*Cckbr*	12426	Chr7: 105074882–105085546	4.14 × 10^−17^	2.382789572	Cholecystokinin B receptor
*Lao1*	100470	Chr4: 118819164–118826107	1.08 × 10^−4^	2.225492179	L-amino acid oxidase 1
*Fosl1*	14283	Chr19: 5497726–5505966	3.59 × 10^−8^	2.074667277	Fos-like antigen 1
*Tph1*	21990	Chr7: 46294065–46321961	5.68 × 10^−8^	2.053908716	Tryptophan hydroxylase 1
*Slc6a4*	15567	Chr11: 76889423–76923169	2.03 × 10^−14^	2.035582069	Solute carrier family 6
*Plin4*	57435	Chr17: 56407587–56416947	3.07 × 10^−165^	1.854500456	Perilipin 4
*Avpr1a*	54140	Chr10: 122284404–122289358	2.46 × 10^−28^	1.797061513	Arginine vasopressin receptor 1A
*Alkal2*	100294583	Chr12: 30934323–30943855	4.04 × 10^−90^	1.777485844	ALK and LTK ligand 2
*Sectm1b*	58210	Chr11: 120944249–120954412	4.27 × 10^−24^	1.65675814	Secreted and transmembrane 1B
*LOC102639021*	102639021	Chr12: 20121571–20189385	1.10 × 10^−13^	1.610514716	–

**Table 3:** Detailed information on the top 20 downregulated DEmRNAs

Downregulated mRNA	Gene ID	Location	*Q* value	Log_2_ fold-change (Oxa/Veh)	Official gene name (NCBI)
*BGIG10090_45075*	BGIG10090_45075	–	5.06 × 10^−27^	−8.020169175	–
*Gm29779*	101056102	Chr5: 23701126–23703111	2.69 × 10^−19^	−7.447276621	–
*Scgb3a2*	117158	Chr18: 43897346–43900464	1.04 × 10^−17^	−7.302543254	Secretoglobin, family 3A, member 2
*Bpifa1*	18843	Chr2: 153984800–153991137	2.74 × 10^−268^	−6.505192561	BPI fold containing family A, member 1
*T*	20997	Chr17: 8653255–8661328	5.40 × 10^−47^	−6.076983553	Brachyury, T-box transcription factor T
*Reg3g*	19695	Chr6: 78443252–78445857	1.68 × 10^−28^	−5.92498046	Regenerating islet-derived 3 gamma
*Serpina1c*	20702	Chr12: 103861185–103871209	3.46 × 10^−6^	−5.578177696	Serine (or cysteine) peptidase inhibitor, clade A, member 1C
*Krt15*	16665	Chr11: 100022585–100026775	1.83 × 10^−5^	−5.376543835	Keratin 15
*Slc32a1*	22348	Chr2: 158449180–158457667	1.95 × 10^−9^	−5.264069106	Solute carrier family 32 (GABA vesicular transporter), member 1
*Usp9y*	107868	ChrY: 1298961–1459782	5.68 × 10^−5^	−5.224540742	Ubiquitin-specific peptidase 9, Y chromosome
*Gabrp*	216643	Chr11: 33500781–33528978	1.77 × 10^−4^	−5.05461574	Gamma-aminobutyric acid (GABA) A receptor, pi
*Muc5b*	74180	Chr7: 141392796–141426826	5.04 × 10^−43^	−4.770822774	Mucin 5, subtype B, tracheobronchial
*Ccl27b*	100040048	Chr4: 42650563–42656008	5.14 × 10^−12^	−4.501215715	Chemokine (C–C motif) ligand 27b
*Gpr17*	574402	Chr18: 32076052–32082925	1.41 × 10^−9^	−4.376543835	G protein-coupled receptor 17
*Olfr287*	634104	Chr15: 98100192–98118937	6.53 × 10^−5^	−4.302543254	Olfactory receptor 287
*Mup2*	17841	Chr4: 60135913–60139977	1.31 × 10^−4^	−4.272662691	Major urinary protein 2
*Sall3*	20689	Chr18: 81010204–81030236	1.10 × 10^−11^	−4.142078582	Spalt-like transcription factor 3
*Pigr*	18703	Chr1: 130754421–130779986	3.56 × 10^−11^	−4.084363084	Polymeric immunoglobulin receptor
*Krt8*	16691	Chr15: 101905146–101912777	8.75 × 10^−25^	−4.067439781	Keratin 8
*Krt18*	16668	Chr15: 101936651–101940461	1.69 × 10^−6^	−3.861970662	Keratin 18

**Table 4:** Detailed information on the top 20 upregulated DElncRNAs

Upregulated lncRNA	Gene ID	*Q* value	Log_2_ fold-change (Oxa/Veh)	Location
*Gm39752*	105244074	2.37 × 10^−8^	6.008024	Chr2: 4225977..4276329
*Gm30259*	102632094	2.51 × 10^−8^	6.002835	Chr18: 38295043..38301092
*Gm7334*	654432	5.53 × 10^−8^	5.934977	Chr17: 51005553..51006920
*BGIG10090_42704*	BGIG10090_42704	1.89 × 10^−4^	4.993272	–
*BGIG10090_44126*	BGIG10090_44126	4.73 × 10^−4^	4.840687	–
*BGIG10090_42484*	BGIG10090_42484	1.22 × 10^−20^	3.772101	–
*Gm30401*	102,632,286	3.10 × 10^−4^	3.267312	Chr12: 27133549..27149088
*Gm13912*	102631868	1.73 × 10^−8^	3.147018	Chr2: 108014061..108121664
*BGIG10090_35415*	BGIG10090_35415	1.78 × 10^−4^	2.656328	–
*BGIG10090_47237*	BGIG10090_47237	2.96 × 10^−6^	2.208419	–
*BC040756*	414073	3.04 × 10^−6^	1.812118	Chr17: 46662573..46668075
*4931408D14Rik*	77059	8.22 × 10^−4^	1.554834	Chr19: 37235822..37241494
*BGIG10090_39118*	BGIG10090_39118	1.06 × 10^−4^	1.440149	–
*Gm31419*	102633638	4.44 × 10^−4^	1.425377	Chr3: 85334574..85399106
*Gm29864*	102631557	2.10 × 10^−6^	1.39708	Chr12: 17713657..17724636
*BGIG10090_36259*	BGIG10090_36259	7.13 × 10^−6^	1.344257	–
*BGIG10090_37941*	BGIG10090_37941	9.33 × 10^−4^	1.243065	–
*BGIG10090_43021*	BGIG10090_43021	1.18 × 10^−4^	1.230786	–
*Gm39698*	105244003	1.50 × 10^−8^	1.230786	Chr1: 169748473..169756950
*BGIG10090_40800*	BGIG10090_40800	1.71 × 10^−4^	1.172729	–

**Table 5:** Detailed information on the top 20 downregulated DElncRNAs

Downregulated lncRNA	Gene ID	*Q* value	Log_2_ fold-change (Oxa/Veh)	Location
*BGIG10090_45547*	BGIG10090_45547	1.03 × 10^−6^	−5.71111	–
*BGIG10090_39783*	BGIG10090_39783	3.24 × 10^−5^	−5.30254	–
*BGIG10090_42558*	BGIG10090_42558	4.66 × 10^−5^	−5.25232	–
*BGIG10090_45353*	BGIG10090_45353	9.13 × 10^−5^	−5.15559	–
*BGIG10090_38159*	BGIG10090_38159	4.03 × 10^−5^	−3.87701	–
*BGIG10090_45356*	BGIG10090_45356	5.56 × 10^−8^	−3.14749	–
*Gm32122*	102634575	8.56 × 10^−6^	−2.59894	Chr8: 97098346..97130726
*Gm38402*	171588	8.14 × 10^−4^	−2.51405	Chr8: 97098346..97130726
*Gm42411*	102640933	7.44 × 10^−4^	−2.33438	Chr1: 42682365..42696565
*BGIG10090_38503*	BGIG10090_38503	5.29 × 10^−4^	−2.17548	–
*BGIG10090_40391*	BGIG10090_40391	1.08 × 10^−7^	−2.10192	–
*Gm31733*	102634054	5.02 × 10^−6^	−1.9477	Chr19: 15630127..15647182
*Gm31102*	102633220	4.62 × 10^−4^	−1.92908	Chr1: 155118875..155132597
*BGIG10090_42607*	BGIG10090_42607	1.00 × 10^−4^	−1.91711	–
*BGIG10090_38251*	BGIG10090_38251	7.24 × 10^−4^	−1.88469	–
*Gm35101*	102638567	4.24 × 10^−4^	−1.86197	Chr10: 99496629..99527876
*BGIG10090_47468*	BGIG10090_47468	1.43 × 10^−4^	−1.82721	–
*Gm41177*	105245784	2.22 × 10^−4^	−1.79158	Chr14: 65409766..65422786
*BGIG10090_34766*	BGIG10090_34766	3.26 × 10^−8^	−1.75137	–
*Gm41004*	105245561	3.76 × 10^−4^	−1.60724	Chr13: 74557105..74564787

We then analyzed the class distribution of DElncRNAs. The DElncRNAs were categorized into four distinct groups, namely, intergenic lncRNAs, sense lncRNAs, antisense lncRNAs, and intronic lncRNAs. Among these DElncRNAs, intergenic lncRNAs belonged to the largest group, containing 241 lncRNAs (56 upregulated and 185 downregulated). The other group of DElncRNAs included 58 antisense lncRNAs (13 upregulated and 45 downregulated), 20 sense lncRNAs (3 upregulated and 17 downregulated), and 55 intronic lncRNAs (5 upregulated and 50 downregulated).

### Bioinformatics analysis of DEmRNAs and DElncRNAs in DRG of ACD model mice

To investigate the mechanisms underlying the scratching response in allergic contact dermatitis, we carried out Gene Ontology (GO) analysis of the DEmRNAs. For upregulated DEmRNAs, we found that the response to positive regulation of secretion, sensory perception of pain, positive regulation of secretion by cell, etc. constitute the mostly enriched biological process (Fig. 2C). Passive transmembrane transporter activity, channel activity, gated channel activity, ion channel activity, etc. constitute the mostly enriched molecular function (Fig. 2E). Proteinaceous extracellular matrix, extracellular matrix, external side of plasma membrane, etc. constitute the most significantly enriched cellular function (Fig. 2G).

Conversely, for downregulated DEmRNAs, the most significantly enriched biological process was extracellular matrix organization, skeletal system development, ossification, extracellular structure organization, etc. (Fig. 2D). The most significantly enriched molecular function of downregulated DEGs was glycosaminoglycan binding and growth factor binding, etc. (Fig. 2F). The most significantly enriched cellular component of downregulated DEGs was extracellular matrix and proteinaceous extracellular matrix, etc. (Fig. 2H).

KEGG analysis was conducted to further analyze these DEmRNAs. As shown in Additional file 7: Fig. S3A, the upregulated DEmRNAs were mainly involved in neuroactive ligand–receptor interaction, extracellular matrix (ECM)–receptor interaction, inflammatory mediator regulation of TRP channels and NOD-like receptor signaling pathway, etc. The downregulated DEmRNAs were mainly involved in ECM–receptor interaction and focal adhesion, etc. (Additional file 7: Fig. S3B).

**Fig. 3:**
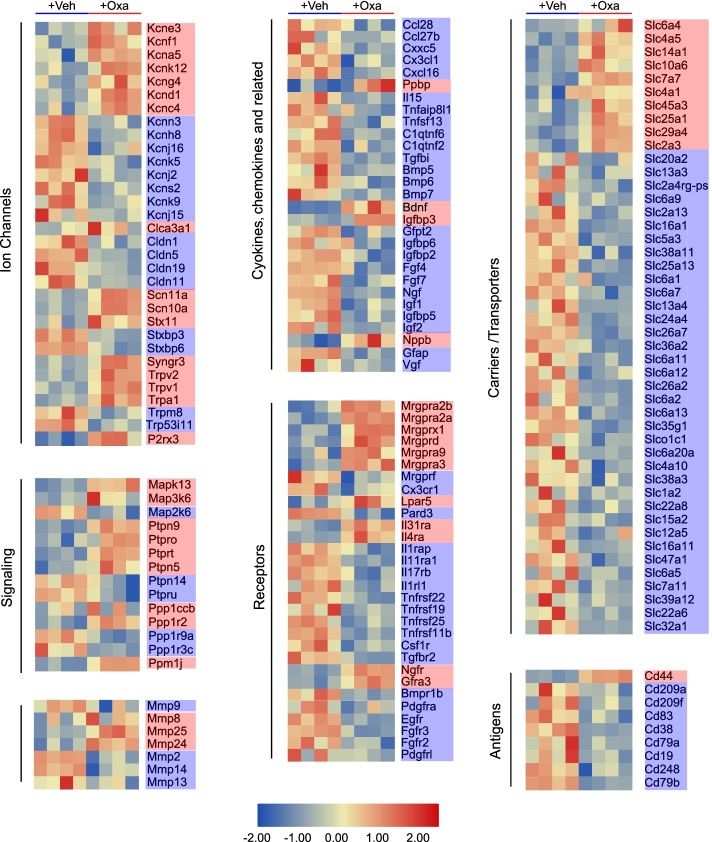
Heatmap summaries of representative DEmNRAs related to sensory neuron signal transduction, pain, itch, and inflammation. Red genes indicate upregulated DEmRNAs, whereas blue genes indicate downregulated DEmRNAs

We further focused on the genes that belong to functional categories related to sensory neuron signal transduction, itch, pain, and inflammation, as well as uncharacterized receptors and ion channels. These genes were laid out in heatmap as shown in Fig. 3. A certain number of these genes, including the well-established itch or pain receptor *Mrgprx1*, *Mrgprd*, *Mrgpra3*, *Il31ra*, and *Il4ra*, and nociceptive ion channels *Scn10a*, *Scn11a*, *Trpv1*, and *Trpa1*, were significantly upregulated in DRG of oxazolone-treated mice versus control mice (Fig. 3). Protein–protein interaction (PPI) network analysis of all upregulated DEGs is shown in Additional file 8: Fig. S4. PPI analysis indicated that the predominant hub genes included *Stat3*, *Timp1*, *Bdnf*, *Gng4*, *Ppbp*, and *CD44* (Additional file 8: Fig. S4).

We then compared our RNA-seq dataset with a recently published RNA-seq dataset. This RNA-seq dataset investigated gene expression changes in trigeminal ganglia of a mouse model of atopic dermatitis that showed skin inflammation and robust pruritus response [16]. Dataset GSE132173 was downloaded from Gene Expression Omnibus. Comparison of our dataset with this published dataset of atopic dermatitis mouse model identified a core set of 39 genes in peripheral sensory neurons that are exclusively affected by skin inflammation (Additional file 9: Table S4). Some of these genes are well known to be involved in itch mechanism, including *Il31ra*, *Nppb*, *Calca*, and *Trpa1*. This analysis could help to identify the core genes that might mediate itch response.

GO enrichment analysis was further performed to study DElncRNA-related DEmRNA genes. Venn diagram indicated there were 53 overlapped genes (Additional file 6: Fig. S2C, Additional file 10: Table S5). GO analysis indicated that the most significantly enriched cellular component of these DElncRNA-related DEmRNA genes included synaptic membrane, synapse, etc. (Additional file 6: Fig. S2D). The most significantly enriched molecular function of DElncRNA-related genes included RNA-directed DNA polymerase activity, endonuclease activity, phosphatidylinositol 3-kinase activity, etc. (Additional file 6: Fig. S2E). The most significantly enriched biological process of DElncRNA-related genes included DNA integration, virion assembly, etc. (Additional file 6: Fig. S2F).

We further performed co-expression analysis of DElncRNAs with target DEmRNAs. We established a co-expression network based upon DElncRNAs and their potential target DEmRNAs (Additional file 11: Fig. S5). We identified some major hub lncRNAs, including *3110039I08Rik*, *1700048O20Rik*, *G630064G18Rik*, *1700123M08Rik*, and *C430002N11Rik*. Of particular note was *B230216N24Rik*, which targets against *Pirt* gene, a well-established gene specifically expressed in peripheral sensory neurons and involved in itch, pain, and TRPV1 modulation [31, 32].

### qPCR validation of DEmRNAs and DElncRNAs

**Fig. 4:**
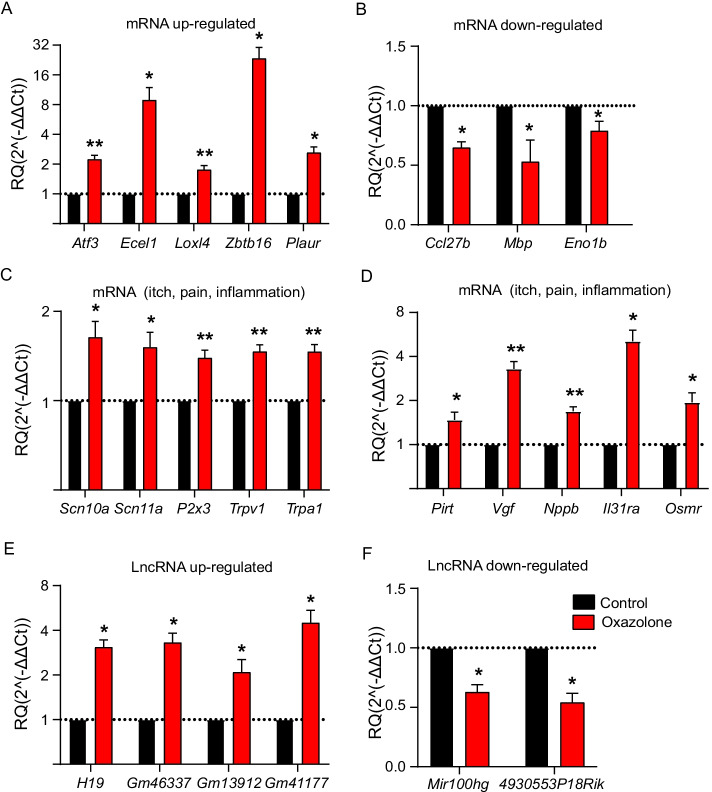
Validation of DEmNRAs and DElncRNAs by qPCR testing. **A** Five randomly picked upregulated DEmRNAs were examined via qPCR testing. **B** qPCR examination of the expression of three randomly picked downregulated DEmRNAs. **C**, **D** qPCR examination of the expression of certain representative DEmRNAs relevant with itch, pain, or inflammation process. **E** qPCR examination of the expression of four randomly selected upregulated DElncRNAs. **F** qPCR examination of the expression of two randomly selected upregulated DElncRNAs. *p < 0.05, **p < 0.01 versus control group. *n* = 6 mice per group

We further verified the RNA-seq data by qPCR testing of a bunch of DEmRNAs as well as DElncRNAs. Eight DEmRNAs (including five up- and three downregulated) and six DElncRNAs (including four up- and two downregulated) were picked for qPCR verification. The results indicated that mRNA expression of *Atf3*, *Ecel1*, *Loxl4*, *Zbtb16*, and *Plaur* was significantly upregulated and that of *Ccl27b*, *Mbp*, and *Eno1b* was significantly downregulated (Fig. 4A, B). The expression change tendencies revealed by qPCR were consistent with RNA-seq. We then focused on genes related to inflammation, itch, and pain. Ten such genes (*Scn10a*, *Scn11a*, *P2x3*, *Trpv1*, *Trpa1*, *Pirt*, *Vgf*, *Nppb*, *Il31ra*, and *Osmr*) were selected and subject to qPCR assay. The results of qPCR assay showed that the expression of all these ten genes was significantly upregulated. These results showed consistency with our RNA-seq results (Fig. 4C, D). Furthermore, as shown in Fig. 4E, F, the results indicated four upregulated (*H19*, *Gm46337*, *Gm13912*, and *Gm41177*) and two downregulated (*Mir100hg* and *4930553P18Rik*) DElncRNAs. These results are consistent with the RNA-seq dataset.

### The expression of the pain and itch receptor MrgprD is functionally upregulated in DRG neurons of oxazolone-induced ACD model mice

**Fig. 5:**
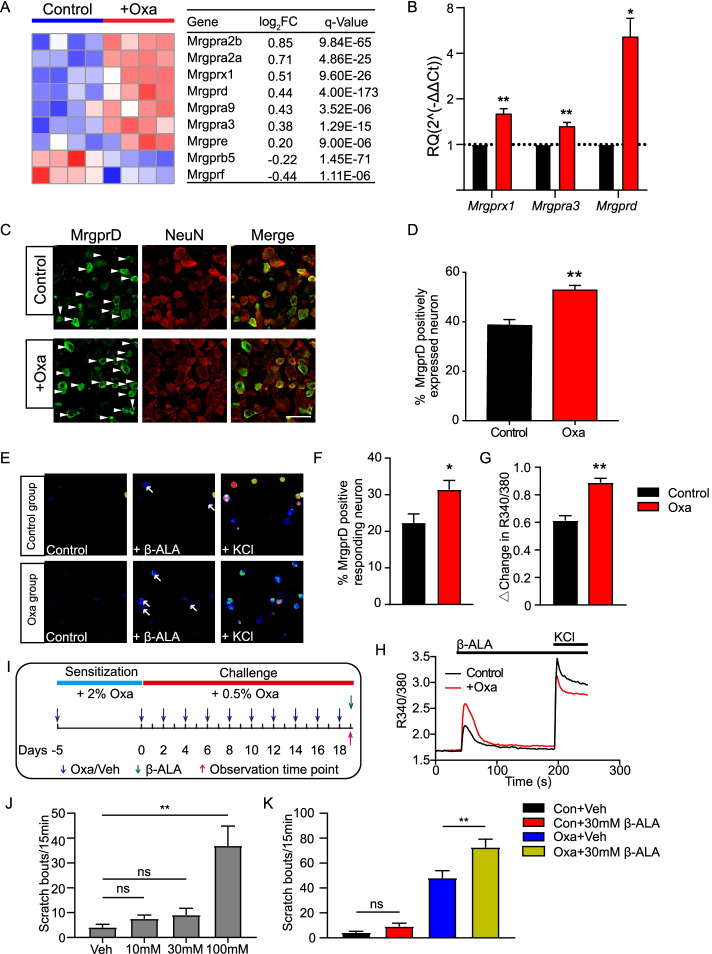
The expression and functional activity of pain- and itch-related receptor MrgprD are upregulated in DRG neurons from oxazolone-induced ACD model mice. **A** Heatmap showing the expression of *Mrgpr* family genes identified in DRG of oxazolone versus control group mice. *n* = 4 mice per group. **B** qPCR validations of the expression of three itch- or pain-related *Mrgpr* genes, namely, *Mrgprx1*, *Mrgpra3*, and *Mrgprd*. *n* = 6–8 mice per group. *p < 0.05, **p < 0.01 versus control group. **C** Representative immunofluorescence images indicating MrgprD antibody staining of DRGs from control and oxazolone group of mice. Scale bar is 50 μm. Areas staining positive for MrgprD are shown in green. NeuN staining (red) was used to illustrate DRG neurons. **D** Summarized percentages of MrgprD^+^ neurons per field. The total neuronal cell numbers were deduced from NeuN^+^ staining. *n* = 6 mice per group. **p < 0.01 versus control group. **E** Pseudo-color images of Fura-2-based ratiometric Ca^2+^ imaging of C1–T1 DRG neurons from oxazolone and control group of mice in response to endogenous MrgprD-specific agonist β-alanine (8 mM). At the end of all recordings, 40 mM KCl was perfused to trace all alive neurons. **F** Summary of the percentages of neurons responding to β-alanine challenge. **G** Summary of Δ increase in peak 340/380 ratio upon β-alanine challenge. *n* = 7 tests per group. Each group included 200–300 neurons. *p < 0.05, **p < 0.01 versus control group. **H** Representative Ca^2+^ traces of DRG neurons in response to β-alanine and KCl from oxazolone and control group of mice. **I** Protocol for behavioral studies. **J** The effect of different dosages of β-alanine (10, 30, and 100 mM in 50 μl) on scratching behaviors in naïve mice. Beta-alanine was subcutaneously injected into the neck of naïve mice. Vehicle group received PBS injection. **p < 0.01 as indicated. NS, no significance. **K** Summary of scratching bouts upon vehicle/β-alanine (30 mM) injection in control or oxazolone group of mice. *n* = 6–7 mice per group

*Mrgprs* belong to a group of G-protein-coupled receptors. Many receptors in this group are specifically distributed in peripheral sensory neurons. The main function of these receptors is to sense and generate itch or pain signals [33, 34]. Therefore, analyzed the gene expression changes of all *Mrgpr* genes in DRG of oxazolone model mice versus control mice by RNA-seq. RNA-seq identified a number of *Mrgprs* genes that showed significant up- or downregulation after oxazolone challenge (Fig. 5A). Among these genes, *Mrgprx1*, *Mrgpra3*, and *Mrgprd* are well-established itch or pain receptors (Fig. 5A). We then performed qPCR to further verify the RNA-seq dataset. We found that the expression of *Mrgprd* showed the highest fold changes among these genes (Fig. 5B). It is known that MrgprD activation can induce pain- and itch-like scratching responses, and the excitability of MrgprD-expressing DRG neurons is significantly increased in a mouse model of ACD [35, 36]. Regarding the important role of MrgprD in mediating chronic pain and itch, we validatedthe protein expression of MrgprD by immunostaining in DRG neurons. Immunostaining showed that the percentage of MrgprD positively expressed DRG neurons among all DRG neurons (stained with NeuN) was significantly increased in oxazolone-challenged mice compared with control mice (Fig. 5C, D). These results indicate that MrgprD expression in DRG neurons is upregulated during ACD condition.

We then set to explore the functional consequences of MrgprD overexpression in DRG neurons of ACD mice. It is known that MrgprD can be activated by endogenous agonist β-alanine to produce Ca^2+^ transients and sensory neuron hyperexcitability, which underlies the cellular mechanisms of β-alanine-induced acute scratching response [36]. The involvement of MrgprD in β-alanine-induced acute scratching is well established. However, the functional changes of MrgprD under chronic ACD condition still remain elusive. Therefore, we performed live cell ratiometric Ca^2+^ imaging on acutely dissociated C1–T1 DRG neurons from both oxazolone-challenged and control mice to monitor functional changes of MrgprD. As reported previously, β-alanine (8 mM) produced Ca^2+^ transients in a certain percentage of DRG neurons from control mice (Fig. 5E, F) [36]. The percentage of DRG neurons responding to β-alanine was significantly higher in oxazolone-challenged group versus control group (Fig. 5E, F). Moreover, the magnitude of the Ca^2+^ transients triggered by β-alanine was greater in oxazolone-challenged group than the corresponding controls (Fig. 5G, H).

We further corroborated the in vitro results with in vivo behavioral studies. We first investigated the concentration of β-alanine that elicited scratching behavior in mice. A series of concentrations of β-alanine (10, 30, and 100 mM) were subcutaneously injected into the neck skin of naïve mice. We found that 10 or 30 mM β-alanine caused only minimal scratching responses, whereas 100 mM β-alanine triggered robust scratching behaviors (Fig. 5J), a result consistent with previous study [36]. We then chose 30 mM β-alanine for our next experiment to see if the scratching response can be potentiated in oxazolone-induced ACD model mice. When 30 mM β-alanine was injected into the neck skin of oxazolone-challenged mice, it began to trigger obvious scratching behaviors in these mice (Fig. 5K). Therefore, these results suggest that the itch and pain receptor MrgprD expression is functionally upregulated in DRG neurons of oxazolone-induced ACD model mice.

## Discussion

In this study, we performed transcriptome profiling to examine the effects of skin inflammation on DRG neurons that innervate the skin of ACD model mice and identified a number of DEmRNAs and DElncRNAs. The expression of these DEGs was further confirmed by qPCR. We examined the molecular functions, cellular component, and biological processes of these DEGs by bioinformatics analysis. We further identified a number of DEGs specifically related to sensory neuron signal transduction, itch, pain, and neuroinflammation. We found that the expression of the pain and itch receptor MrgprD is functionally upregulated in DRG neurons of ACD model mice, which may contribute to peripheral pain and itch sensitization mechanisms during ACD.

Oxazolone-induced chronic ACD model mice developed remarkable scratching behavior towards the challenged site. Our recent study revealed that the scratching behavior induced by oxazolone was actually due to a mixed itch and pain sensation using the classical “cheek model” [13, 37]. We showed that repetitive oxazolone challenge on mouse cheek provokes both scratching (with hindpaw) and wiping (with forepaw) behavior, which could be interpreted as itch and pain sensation, respectively [13]. Consistent with our findings, studies from LaMotte’s lab also found that the contact sensitizer squaric acid dibutyl ester-induced ACD model mice developed similar itch- and pain-related behaviors [2, 38]. These studies support the notion that ACD model mice developed a mixed itch and pain sensation. This phenomenon is consistent with studies from human volunteers showing a mixed itch and pain sensation in an experimental model of ACD [4].

Recently, much progress has been made to elucidate the pain and itch mechanisms of ACD. One of our studies identified important contributions of neuronal TRPA1 to skin inflammation as well as scratching response in oxazolone-induced mouse ACD model [12]. We further demonstrate that IL-33 acts on receptor ST2 expressed in peripheral DRG neurons to trigger neuronal hyperexcitability and scratching response in a mouse poison-ivy-induced ACD model [14]. ST2 receptor’s function in DRG neurons is upregulated under skin inflammation, which in turn enhanced IL-33’s effect on DRG neurons [14]. Other studies demonstrate that ACD upregulates CCL2 and CXCL10 signaling in a subpopulation of cutaneous small-diameter DRG neurons and that CCL2 and CXCL10 can activate these neurons through neuronal receptors to elicit itch- and pain-like behavior [15, 38]. Thus, skin inflammation can affect the function of peripheral sensory neurons innervating the local skin, resulting in enhanced pain and itch sensation.

To gain a comprehensive view of how skin inflammation may affect sensory neuron function, we performed transcriptome RNA-seq of DRG innervating the inflamed skin. GO analyses identified that one of the mostly enriched biological processes of upregulated genes in DRG of the oxazolone versus control group was relevant with sensory perception of pain. Some of the key genes allocated to this category include *Trpa1*, *Trpv1*, *5-Htr2a*, and *Ngfr*. TRPA1 is involved in mediating pain and histamine-independent itch [14, 39–43]. In addition, TRPA1 also contributes to chronic itch, including animal models of ACD, atopic dermatitis, and dry skin condition [12, 44, 45]. In contrast, TRPV1 is well recognized for mediating pain and histamine-dependent itch [46, 47]. Our previous study using the same oxazolone-induced ACD mouse model identified that genetic knockout or pharmacological blockage of TRPA1 or 5-HT_2A_R significantly relieved the scratching behavior of model mice [12, 13]. Our present study further found that *Trpa1* and *5-Htr2a* expression was upregulated in DRG in the context of ACD. Therefore, these studies suggest that TRPA1 or 5-HT2AR may be potential targets for ameliorating pain and itch in ACD.

We previously found that nerve growth factor (NGF) concentration was significantly increased in the inflamed skin of oxazolone-induced ACD model mice [12]. Here we further found that the mRNA expression of its receptor Ngfr is significantly increased in DRG innervating the inflamed skin. NGF is a neurotrophic factor that is critical for regulating the growth, maintenance, and survival of neurons. NGF acts on two receptors, namely, the high-affinity TrkA and low-affinity receptor p75 (NGFR), to initiate biological functions. The expression of NGF has been reported to be increased in the inflamed skin of several chronic itch conditions, including psoriasis and prurigo nodularis [48, 49]. The release of NGF in inflamed tissues can enhance the innervation and functional activity of peripheral sensory neurons [50]. Thus, NGF may act via its receptors in DRG neurons to promote innervation and activity of sensory neurons, which contributes to potentiation of itch or pain sensation in ACD.

Many of the Mrgprs exhibited preferential distribution exclusively in peripheral sensory neurons. Several of the Mrgprs have been identified to be receptors for itch and pain, including MrgprA3, MrgprC11, and MrgprD [33, 51]. In this study, we screened the gene expression of all members of the Mrgprs family in DRG from ACD model mice by RNA-seq and found that *Mrgpra3*, *Mrgprc11*, and *Mrgprd* showed significant upregulation. qPCR indicated that *Mrgprd* showed the highest fold increase among these three genes. We further found that the protein expression of MrgprD was also significantly upregulated in DRG neurons of ACD model mice versus control mice. Ca^2+^ imaging showed that β-alanine-induced MrgprD activation was enhanced in DRG neurons from ACD model mice. Behavioral assay further confirmed that β-alanine-induced scratching behavior was enhanced in ACD model mice. MrgprD is involved in both pain and itch mechanism [36, 52]. Intake of β-alanine induces skin paresthesia among volunteers, including pins-and-needles, tingling, and itch sensations, indicating that β-alanine produces both itch and pain sensation [53]. These results indicated that MrgprD may contribute to pain and itch sensitization in ACD. However, it should be noted that, although the changes of MrgprD expression are robust, other pain- or itch-related receptors (as mentioned above) may also be involved. For example, Su et al. recently presented the proteome profiling of trigeminal ganglion of a mouse model of ACD and identified that complement component 3 in trigeminal satellite cells contributed to itch and pain sensation in ACD mice [54]. Therefore, a diversity of pain and itch mediators and signaling could together contribute to the overall scratching response of ACD, which reflects the complexity of the mechanism involved. Further functional validations of these potential pain- or itch-related genes are needed to unravel the detailed mechanisms underlying ACD.

LncRNAs belong to a category of RNAs with length longer than 200 nucleotides, but with no protein coding functions [21]. They may contribute to protein functional modulations or transcriptional regulations by acting upon DNA, RNA, and proteins. Mounting evidence suggest that dysregulated expression of lncRNAs took place in injured nerves, DRG, and spinal cord, following peripheral nerve injury. These lncRNAs contribute to chronic pain by regulating pain-related gene expression in the sensory nerve system [55–57]. However, to date, studies related to lncRNA identification in peripheral sensory ganglia under chronic itch conditions are still lacking. Therefore, we explored the potential lncRNAs and identified their biological functions in DRG of ACD model mice. KEGG analysis of DElncRNAs identified that phosphatidylinositol 3-kinase (PI3K) activity is one of the most significantly enriched pathway in molecular function. PI3K is well recognized for its involvement in chronic pain modulation [58]. In addition, PI3K played a critical role in mediating acute itch transmission by GRP/GRPR signaling or trypsin [59, 60]. Spinal PI3K is also involved in mediating dry-skin-induced chronic itch condition [59], but the exact contribution of PI3K in DRG to pain and itch in ACD condition remains unknown. Therefore, the role of PI3K and its potential modulation by lncRNAs in DRG during ACD should be explored.

It should be noted that our study has some limitations: (1) only male mice were used in the present study. It is known that sex may play a role in pain and itch [61, 62]. Therefore, it would be interesting to test β-alanine-induced scratching response in female ACD mice as well, which may help to extend the significance of our current findings. (2) Retrograde labeling was not applied in Ca^2+^ imaging or immunostaining of DRG neurons. Retrograde labeling can label skin-innervating DRG neurons, which helps to focus more specifically on this specific group of sensory neurons. (3) MrgprD genetic knockout mice or knockdown by small interfering RNA (siRNA) was not used in the present study, although these techniques could help to confirm the role of MrgprD in ACD-induced scratching behavior. Therefore, further efforts will be needed to address these issues.

## Conclusions

Our study presented a detailed transcriptome profiling of peripheral DRG innervating the inflamed skin of a mouse model of ACD. Our study suggested that the pain and itch receptor MrgprD might contribute to peripheral pain and itch sensitization during ACD. Thus, targeting MrgprD may be an effective method for alleviating itch and pain sensation in ACD.

## Supplementary Information

**Additional file 1.** Supplementary material and methods.

**Additional file 2.** **Table S1.** Read count of all mRNAs.

**Additional file 3.** **Table S2.** Read count of all lncRNAs.

**Additional file 4.** **Table S3.** Sequences of the primers used for qPCR validation of RNA-seq data.

**Additional file 5.** **Fig. S1.** Quality inspection report of total RNA extracted from C1–T1 bilateral DRGs.

**Additional file 6.** **Fig. S2.** Bioinformatics analysis of lncRNAs from oxazolone-induced mouse ACD model. (A) Volcano plot showing lncRNA gene expression profiles. Red and blue spots indicate up- and downregulated DEGs, respectively. Gray spots indicate non-DEGs. (B) Heat map of hierarchical clustering of DElncRNAs of oxazolone group versus control group. (C) Venn diagram indicating the overlapped DElncRNAs-related genes with DEmRNAs. (D–F) GO enrichment analysis including cellular component, molecular function, and biological process of all 53 DElncRNA-related DEmRNAs.

**Additional file 7.** **Fig. S3.** KEGG pathway analysis of DEGs. (A) Bubble plots showing the top ten significant pathways for upregulated DEGs. (B) Bubble plots showing the top ten significant pathways for downregulated DEGs. Larger bubbles indicate higher number of genes. The color of each bubble reflects significance (q-value).

**Additional file 8.** **Fig. S4.** PPI network analysis of all upregulated DEGs. Larger circles and deeper colors reflect more interactions and vice versa.

**Additional file 9.** **Table S4.** Overlapping of DEGs from oxazolone-induced mouse model with mouse model of MC903.

**Additional file 10.** **Table S5.** Overlapping of genes from DElncRNAs-related genes with DEmRNAs.

**Additional file 11.** **Fig. S5.** The co-expression network analysis of DElncRNAs with the potential target DEmRNAs. The red and blue shapes showed DEmRNAs and DElncRNAs, respectively.

## Data Availability

The data supporting the findings of this study are available in this article and its additional files.

## Abbreviations

RNA-Seq: RNA sequencing
DRG: Dorsal root ganglia
qPCR: Real‑time quantitative PCR
ACD: Allergic contact dermatitis
LncRNA: Long noncoding RNA
DEG: Differentially expressed gene
KEGG: Kyoto Encyclopedia of Genes and Genomes
GO: Gene Ontology
