# Supplementary material for: Exploring neuronal mechanisms involved in the scratching behavior of a mouse model of allergic contact dermatitis by transcriptomics
Source: Cell Mol Biol Lett. 2022 Feb 19;27:16. doi: 10.1186/s11658-022-00316-w (PMC8903649; doi:10.1186/s11658-022-00316-w)
Supplement: Supplementary file 1 — Additional file 1. Supplementary material and methods. [file 11658_2022_316_MOESM1_ESM.docx]

**Supplementary Material and methods**

*Bioinformatics analysis and data deposition*

The procedures have been described in our previous study [1]. Briefly, the sequencing data was filtered with SOAPnuke (v1.5.2) by (1) Removing reads containing sequencing adapter; (2) Removing reads whose low-quality base ratio (base quality less than or equal to 5) is more than 20%; (3) Removing reads whose unknown base ('N' base) ratio is more than 5%, afterwards clean reads were obtained and stored in FASTQ format. The clean reads were mapped to the reference genome using HISAT2 (v2.0.4). After that, Ericscript (v0.5.5) and rMATS (V3.2.5) were used to detect fusion genes and differential splicing genes (DSGs), respectively. Bowtie2 (v2.2.5) was applied to align the clean reads to the gene set, a database built by BGI, in which known and novel, coding and noncoding transcripts were included. Then expression level of gene was calculated by RSEM (v1.2.12). The heatmap was drawn by pheatmap (v1.0.8) according to the gene expression in different samples. The transcriptome RNA-Seq containing both mRNA and lncRNA dataset was provided as Suppl. Table 1&2 as supplementary materials.

*Protein-protein interaction (PPI) network analysis*

The search tool for the retrieval of interacting genes (STRING) is used to provide information regarding predicted and experimental interactions of proteins and the prediction method of this database is from neighborhood, gene fusion, co-occurrence, co-expression experiments, databases, and text mining. By setting the Combination score > 0.4 as the reliability threshold value, the web based STRING database (http://string-db.org/) was used to produce PPI predictions after uploading the union gene list to the search bar. Based on the interplayed relationships, a PPI network was established and then visualized using the Cytoscape software. The connectivity degree of each protein, namely the number of proteins it connected, was calculated to evaluate its importance in this network.

**References**

[1] Chen R, Yin C, Hu Q, Liu B, Tai Y, Zheng X, Li Y, Fang J, Liu B: Expression profiling of spinal cord dorsal horn in a rat model of complex regional pain syndrome type-I uncovers potential mechanisms mediating pain and neuroinflammation responses. *J Neuroinflammation* 2020, 17:162.
