# Supplementary material for: Exploring neuronal mechanisms involved in the scratching behavior of a mouse model of allergic contact dermatitis by transcriptomics
Source: Cell Mol Biol Lett. 2022 Feb 19;27:16. doi: 10.1186/s11658-022-00316-w (PMC8903649; doi:10.1186/s11658-022-00316-w)
Supplement: Supplementary file 4 — Additional file 4. Table S3. Sequences of the primers used for qPCR validation of RNA-seq data. [file 11658_2022_316_MOESM4_ESM.docx]

**Suppl. Table. 3 Sequences of the primers used for qPCR validation of RNA-Seq data**

| Gene name | | Gene ID | Primer sequence (5’ to 3’) | Amplicon size (bp) |
| --- | --- | --- | --- | --- |
| *β-actin* | 11461 | | F:5’-GTGCTATGTTGCTCTAGACTTCG-3’ | 174 |
|  |  |  | R:5’-ATGCCACAGGATTCCATACC-3’ |  |
| *Scn10a* | 20264 | | F:5’-CAACAATTGCAGAAATCGAAGC-3’ | 162 |
|  |  |  | R:5’-GTCTTCTCTCATTGGCGTTTTT-3’ |  |
| *Scn11a* | 24046 | | F:5’-CATCGTCTTTGTGCTGATCATG-3’ | 89 |
|  |  |  | R:5’-CCTCATTGCTGAAGGAATTGAG-3’ |  |
| *P2x3* | 228139 | | F:5’-AAAGCTGGACCATTGGGATCA-3’ | 107 |
|  |  |  | R:5’-CGTGTCCCGCACTTGGTAG-3’ |  |
| *Trpv1* | 193034 | | F:5’-CCCGGAAGACAGATAGCCTGA-3’ | 92 |
|  |  |  | R:5’-TTCAATGGCAATGTGTAATGCTG-3’ |  |
| *Trpa1* | 277328 | | F:5’-GTCCAGGGCGTTGTCTATCG-3’ | 163 |
|  |  |  | R:5’-CGTGATGCAGAGGACAGAGAT-3’ |  |
| *Mrgprx1* | 404242 | | F:5’-TCTCATCCCACGACACAGAAT-3’ | 86 |
|  |  |  | R:5’-AGCCAGAGTACAATGGTGTTTC-3’ |  |
| *Mrgpra3* | 233222 | | F:5’-CTCAAGTTTACCCTACCCAAAGG-3’ | 233 |
|  |  |  | R:5’-CCGCAGAAATAACCATCCAGAA-3’ |  |
| *Mrgprd* | 211578 | | F:5’-TTTTCAGTGACATTCCTCGCC-3’ | 118 |
|  |  |  | R:5’-GCACATAGACACAGAAGGGAGA-3’ |  |
| *Pirt* | 193003 | | F:5’-GGTTGGATGCCGTGTCTCTGC-3’ | 85 |
|  |  |  | R:5’-TGGTGCTGTGGATGTTGCTCTTG-3’ |  |
| *Vgf* | 381677 | | F:5’-AAGGATGACGGCGTACCAGA-3’ | 114 |
|  |  |  | R:5’-TGCCTGCAACAGTACCGAG-3’ |  |
| *Nppb* | 18158 | | F:5’-TGCTGGAGCTGATAAGAGAAAA-3’ | 96 |
|  |  |  | R:5’-GAAGGACTCTTTTTGGGTGTTC-3’ |  |
| *Il31ra* | 218624 | | F:5’-GCGAATGAGTATGTGACCTCC-3’ | 145 |
|  |  |  | R:5’-CCAATTCTGAGTACGCCTCGT-3’ |  |
| *Osmr* | 18414 | | F:5’-TCTGGATCCAGACACAAAGTAC-3’ | 181 |
|  |  |  | R:5’-CCAGAATAAAGTCACAATGCGT-3’ |  |
| *Atf3* | 11910 | | F:5’-TTGTCAAGGAAGAGCTGAGATT-3’ | 221 |
|  |  |  | R:5’-GCACTCTGTCTTCTCCTTTTTC-3’ |  |
| *Ecel1* | 13599 | | F:5’-CTTCCCACAGTCTCTGAACTAC-3’ | 191 |
|  |  |  | R:5’-AGACGGTGAAGTTGTCATAGAG-3’ |  |
| *Loxl4* | 67573 | | F:5’-GCCAACGGACAGACCAGAG-3’ | 139 |
|  |  |  | R:5’-CCAGGTCAAGGCTGACTCAAA-3’ |  |
| *Zbtb16* | 235320 | | F:5’-CGCCACCTTCGCTCACATACAG-3’ | 142 |
|  |  |  | R:5’-TCTTGCCACAGCCATTACACTCATAG-3’ |  |
| *Plaur* | 18793 | | F:5’-GAACAATACCCTTGGGTGTTC-3’ | 126 |
|  |  |  | R:5’-CAGCCTCTTACGGTATAACTCC-3’ |  |
| *Ccl27b* | 100040048 | | F:5’-ATCCGTGGAACAAGACTAAGCAGAAG-3’ | 122 |
|  |  |  | R:5’-CATGTGGACAATCCTCCTCAGCAG-3’ |  |
| *Cxcl16* | 66102 | | F:5’-CTGGAAGTTGTTCTTGTGATCG-3’ | 128 |
|  |  |  | R:5’-CTGCAACTGGAACCTGATAAAG-3’ |  |
| *Mbp* | 17196 | | F:5’-GACCCAAAGAATAACTGGCAAG-3’ | 157 |
|  |  |  | R:5’-GGTCTTCTTGGATGGTCTGAAG-3’ |  |
| *Eno1b* | 433182 | | F:5’-CATCCACACCTGACCACCAGAATC-3’ | 118 |
|  |  |  | R:5’-AGTTCCGAGGGCTCCAGACAC-3’ |  |
| *H19* | 14955 | | F:5’-TCCTTGGAGACAGTGGCAGAGAC-3’ | 135 |
|  |  |  | R:5’-AATTACGGTGGGTGGGATGTTGTG-3’ |  |
| *Gm46337* | 105246334 | | F:5’-TGGCTGGAGTTGTAGGAGATGAGG-3’ | 84 |
|  |  |  | R:5’-TGCTACGGACAGGTCACCACTAG-3’ |  |
| *Gm13912* | 102631868 | | F:5’-GCAGAGGCAATAAACATGGCTTGG-3’ | 103 |
|  |  |  | R:5’-AACAATGAGGAAGCAGCTCAGGTC-3’ |  |
| *Gm41177* | 105245784 | | F:5’-AGCCGTGACTAGAGCGAGAAGG-3’ | 108 |
|  |  |  | R:5’-CCGTGAACTGTGACCTGAACTGG-3’ |  |
| *Mir100hg* | 73144 | | F:5’-TAAGCAGCAGACGGGCAGAGG-3’ | 101 |
|  |  |  | R:5’-GACACACAGACAGCGGTTCCAAG-3’ |  |
| *4930553P18Rik* | 75355 | | F:5’-GTCCCGCTGCTACACTTGTATTGG-3’ | 99 |
|  |  |  | R:5’-AGCAAGGCACCAGGAGAGTCTATC-3’ |  |
