# Supplementary figures and images for: Exploring neuronal mechanisms involved in the scratching behavior of a mouse model of allergic contact dermatitis by transcriptomics
Source: Cell Mol Biol Lett. 2022 Feb 19;27:16. doi: 10.1186/s11658-022-00316-w (PMC8903649; doi:10.1186/s11658-022-00316-w)

C1-T1 bilateral DRGs

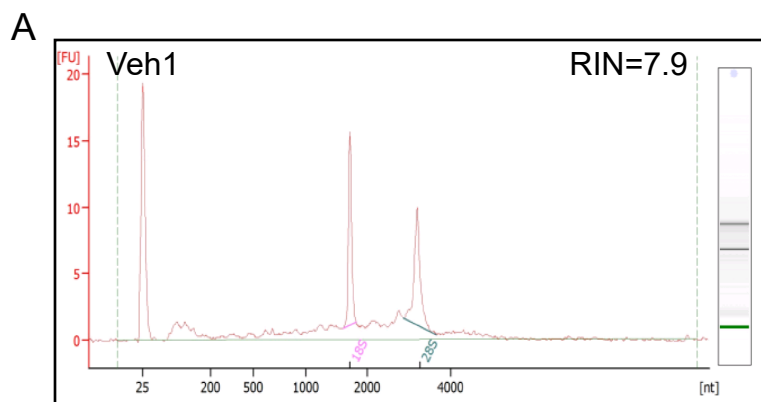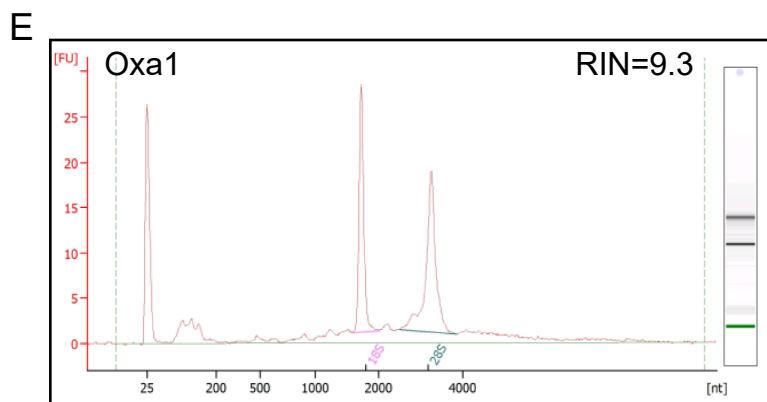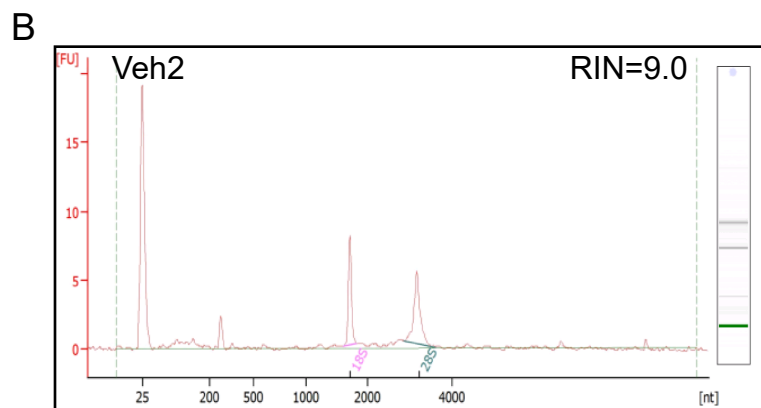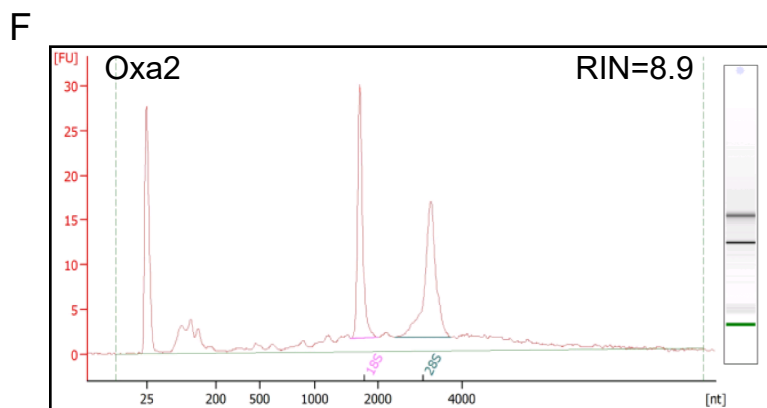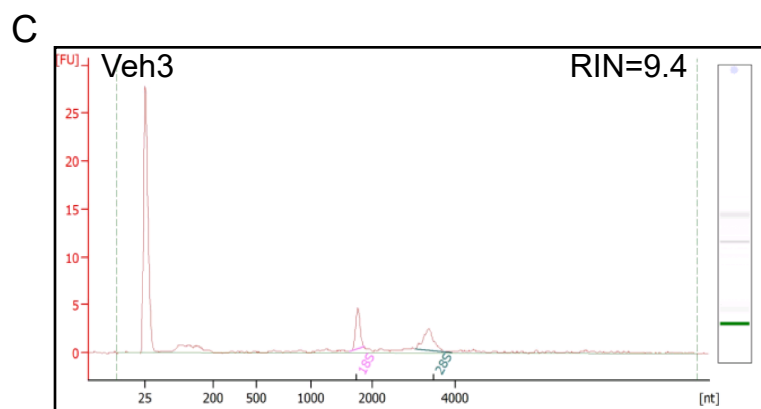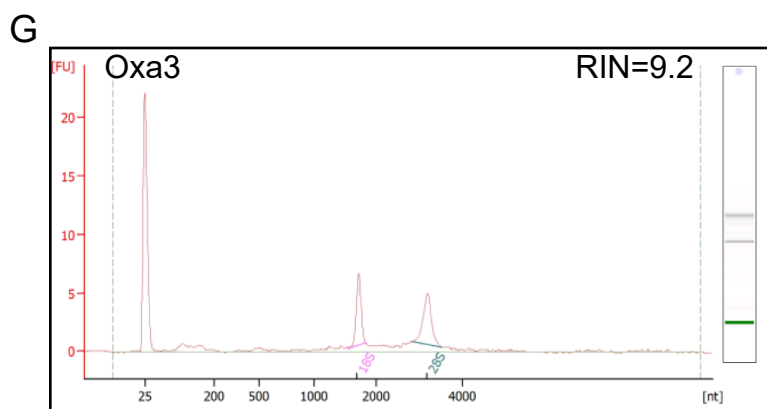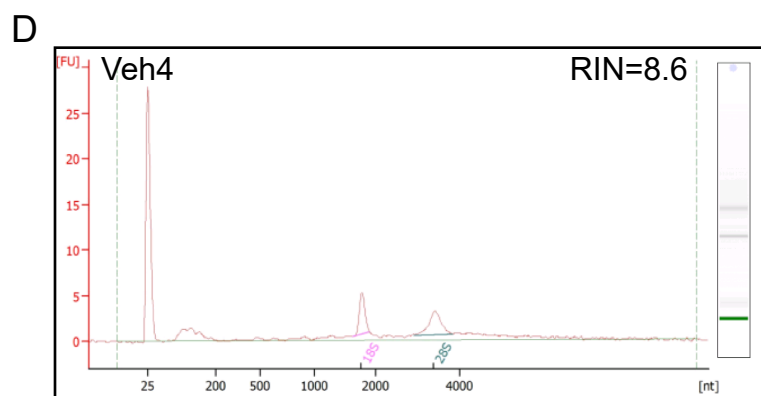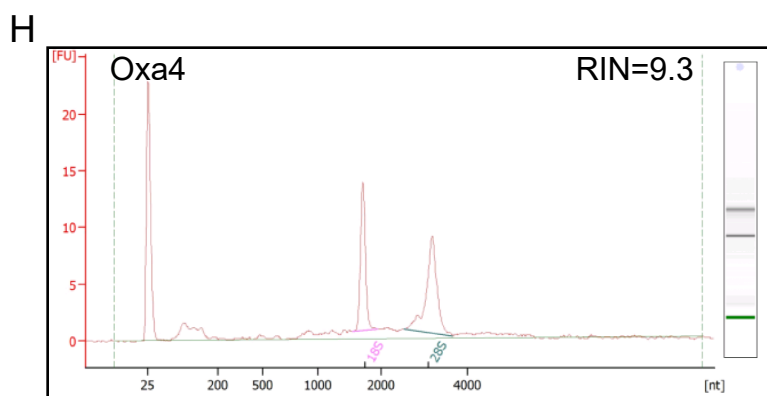

Supplement: Supplementary file 5 — Additional file 5. Fig. S1. Quality inspection report of total RNA extracted from C1–T1 bilateral DRGs. [file 11658_2022_316_MOESM5_ESM.pdf]

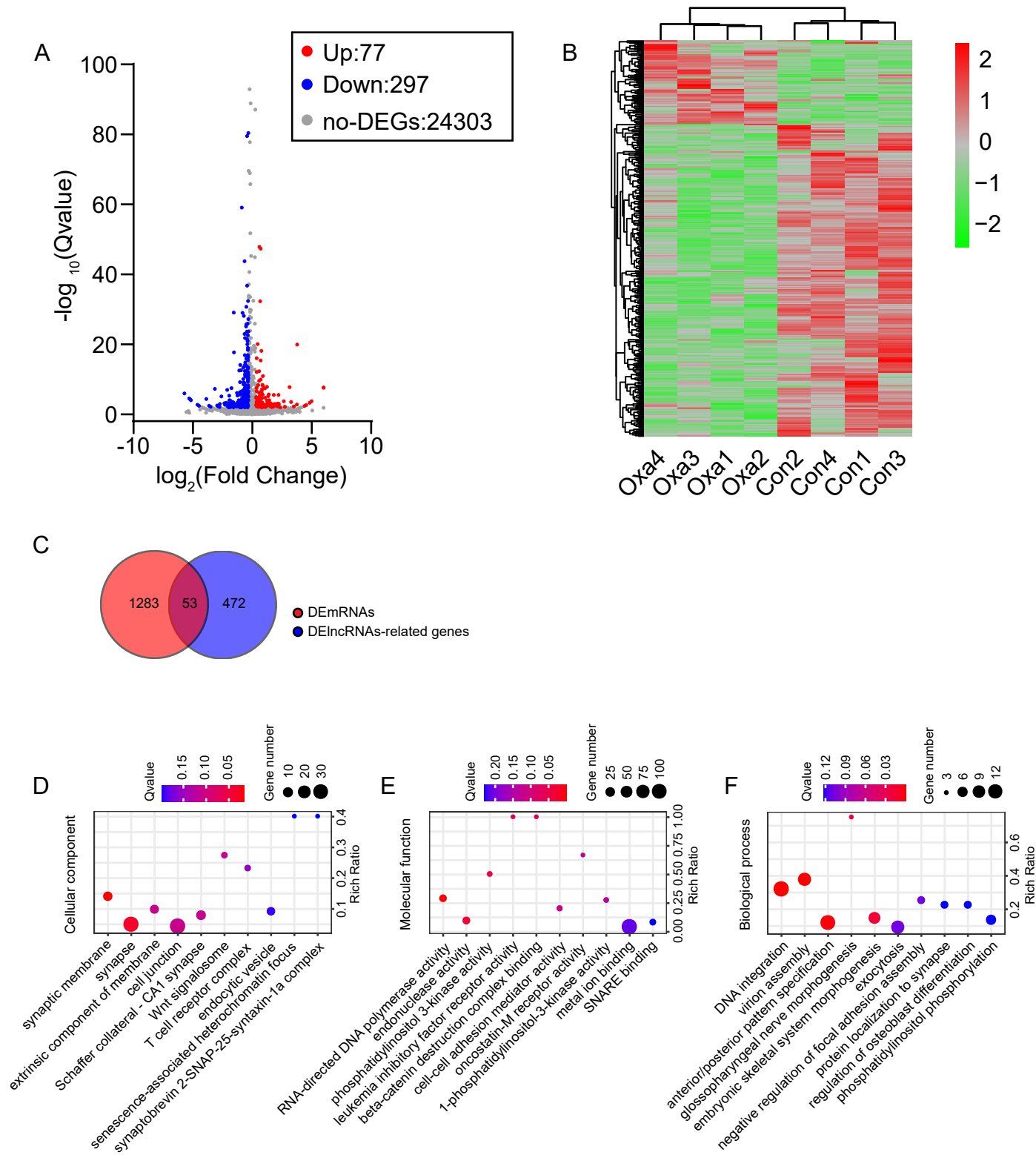

Supplement: Supplementary file 6 — Additional file 6. Fig. S2. Bioinformatics analysis of lncRNAs from oxazolone-induced mouse ACD model. (A) Volcano plot showing lncRNA gene expression profiles. Red and blue spots indicate up- and downregulated DEGs, respectively. Gray spots indicate non-DEGs. (B) Heat map of hierarchical clustering of DElncRNAs of oxazolone group versus control group. (C) Venn diagram indicating the overlapped DElncRNAs-related genes with DEmRNAs. (D–F) GO enrichment analysis including cellular component, molecular function, and biological process of all 53 DElncRNA-related DEmRNAs. [file 11658_2022_316_MOESM6_ESM.pdf]

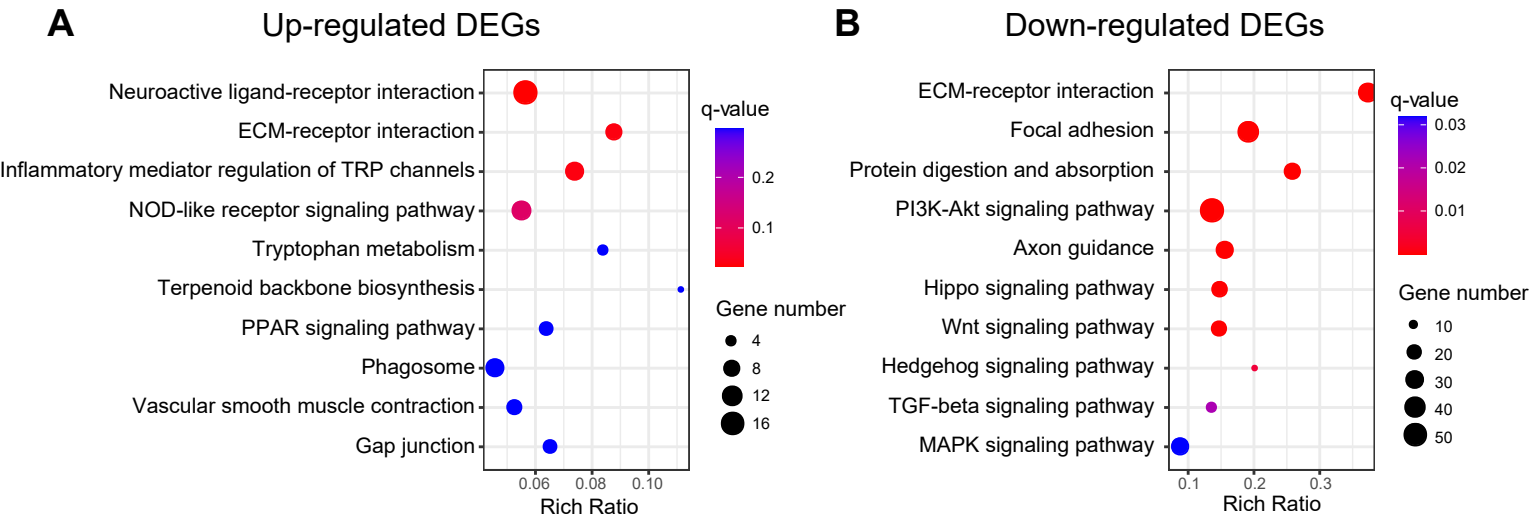

Supplement: Supplementary file 7 — Additional file 7. Fig. S3. KEGG pathway analysis of DEGs. (A) Bubble plots showing the top ten significant pathways for upregulated DEGs. (B) Bubble plots showing the top ten significant pathways for downregulated DEGs. Larger bubbles indicate higher number of genes. The color of each bubble reflects significance (q-value). [file 11658_2022_316_MOESM7_ESM.pdf]

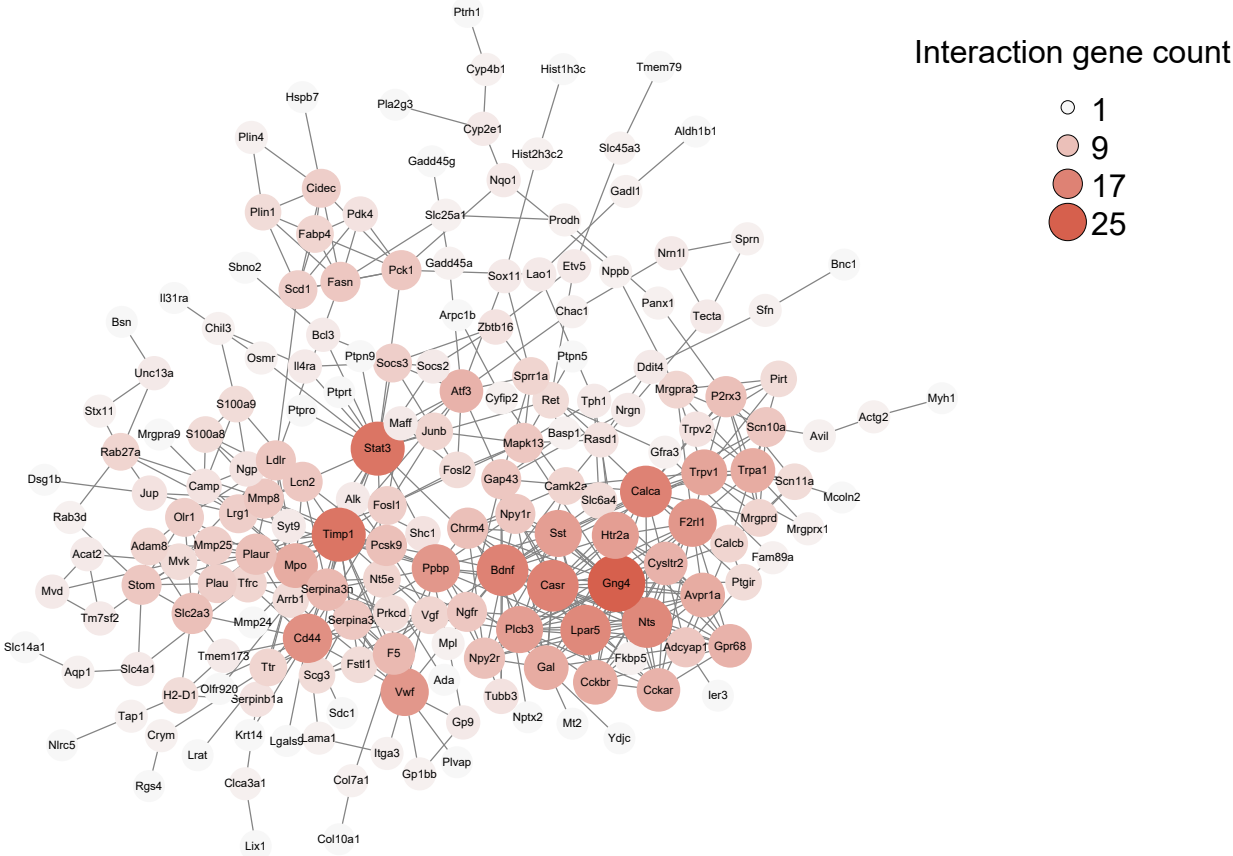

Supplement: Supplementary file 8 — Additional file 8. Fig. S4. PPI network analysis of all upregulated DEGs. Larger circles and deeper colors reflect more interactions and vice versa. [file 11658_2022_316_MOESM8_ESM.pdf]

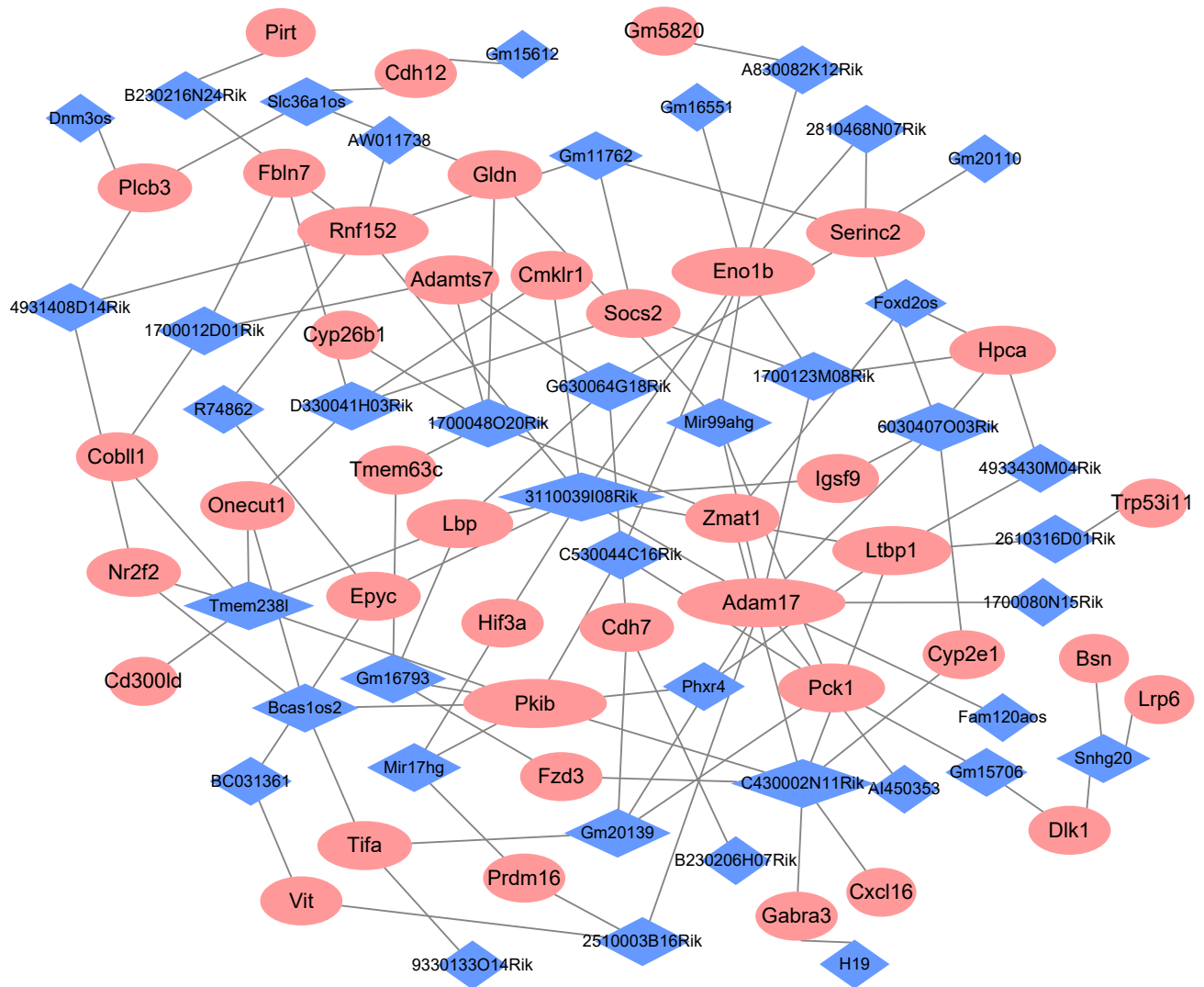

Supplement: Supplementary file 11 — Additional file 11. Fig. S5. The co-expression network analysis of DElncRNAs with the potential target DEmRNAs. The red and blue shapes showed DEmRNAs and DElncRNAs, respectively. [file 11658_2022_316_MOESM11_ESM.pdf]
