# Supplementary material for: Exploring neuronal mechanisms involved in the scratching behavior of a mouse model of allergic contact dermatitis by transcriptomics
Source: Cell Mol Biol Lett. 2022 Feb 19;27:16. doi: 10.1186/s11658-022-00316-w (PMC8903649; doi:10.1186/s11658-022-00316-w)
Supplement: Supplementary file 10 — Additional file 10. Table S5. Overlapping of genes from DElncRNAs-related genes with DEmRNAs. [file 11658_2022_316_MOESM10_ESM.docx]

**Suppl. Table. 5 The detailed information of 53 overlapped genes**

| Gene ID | Gene symbol | Log2 fold change (Oxa/Veh) | regulation | Pathway |
| --- | --- | --- | --- | --- |
| 381677 | Vgf | 1.493269249 | up |  |
| 53417 | Hif3a | 1.424910485 | up |  |
| 211550 | Tifa | 0.711695178 | up |  |
| 18768 | Pkib | 0.700362477 | up |  |
| 268709 | Fam107a | 0.533900594 | up |  |
| 18534 | Pck1 | 0.516653281 | up | Pyruvate metabolism  Glycolysis / Gluconeogenesis  Biosynthesis of antibiotics  Microbial metabolism in diverse environments  Metabolic pathways  Adipocytokine signaling pathway  AMPK signaling pathway  Proximal tubule bicarbonate reclamation  PPAR signaling pathway  Citrate cycle (TCA cycle)  Glucagon signaling pathway  Biosynthesis of secondary metabolites  FoxO signaling pathway  PI3K-Akt signaling pathway  Insulin signaling pathway |
| 230779 | Serinc2 | 0.490609003 | up |  |
| 216233 | Socs2 | 0.465336769 | up | Insulin signaling pathway  Prolactin signaling pathway  Jak-STAT signaling pathway |
| 13106 | Cyp2e1 | 0.461365298 | up | Arachidonic acid metabolism  Metabolism of xenobiotics by cytochrome P450  Drug metabolism - other enzymes  Drug metabolism - cytochrome P450  Steroid hormone biosynthesis  Linoleic acid metabolism  Metabolic pathways |
| 73748 | Gadl1 | 0.450307262 | up | Biosynthesis of secondary metabolites  Pantothenate and CoA biosynthesis  beta-Alanine metabolism  Taurine and hypotaurine metabolism  Metabolic pathways |
| 18053 | Ngfr | 0.408009312 | up | Rap1 signaling pathway  Ras signaling pathway  Neurotrophin signaling pathway  PI3K-Akt signaling pathway  Apoptosis - multiple species  MAPK signaling pathway  Cytokine-cytokine receptor interaction |
| 12217 | Bsn | 0.351409745 | up |  |
| 193003 | Pirt | 0.330921692 | up |  |
| 18797 | Plcb3 | 0.330603111 | up | Gastric acid secretion  Insulin secretion  Chemokine signaling pathway  Calcium signaling pathway  Adrenergic signaling in cardiomyocytes  GnRH signaling pathway  Endocrine and other factor-regulated calcium reabsorption  Vascular smooth muscle contraction  Dopaminergic synapse  Oxytocin signaling pathway  Aldosterone synthesis and secretion  Gap junction  Inositol phosphate metabolism  Glutamatergic synapse  Relaxin signaling pathway  Melanogenesis  Sphingolipid signaling pathway  Serotonergic synapse  Glucagon signaling pathway  Long-term potentiation  Cholinergic synapse  Thyroid hormone signaling pathway  Retrograde endocannabinoid signaling  Cortisol synthesis and secretion  Platelet activation  Pancreatic secretion  Parathyroid hormone synthesis, secretion and action  Estrogen signaling pathway  Renin secretion  Phospholipase D signaling pathway  Thyroid hormone synthesis  Circadian entrainment  Apelin signaling pathway  Long-term depression  Wnt signaling pathway  Phototransduction – fly  Rap1 signaling pathway  Phosphatidylinositol signaling system  Inflammatory mediator regulation of TRP channels  Salivary secretion  NOD-like receptor signaling pathway  Metabolic pathway  cGMP-PKG signaling pathway |
| 217733 | Tmem63c | 0.329237148 | up |  |
| 68075 | Lurap1 | -0.324911067 | down |  |
| 215693 | Zmat1 | -0.327026963 | down |  |
| 16803 | Lbp | -0.338659358 | down | Toll-like receptor signaling pathway  NF-kappa B signaling pathway |
| 14365 | Fzd3 | -0.340612183 | down | Wnt signaling pathway  Axon guidance  Melanogenesis  Hippo signaling pathway  Signaling pathways regulating pluripotency of stem cells  mTOR signaling pathway |
| 70673 | Prdm16 | -0.340682439 | down | Thermogenesis |
| 11819 | Nr2f2 | -0.344323848 | down |  |
| 215654 | Cdh12 | -0.347906472 | down |  |
| 108153 | Adamts7 | -0.355438976 | down |  |
| 14396 | Gabra3 | -0.361366737 | down | Retrograde endocannabinoid signaling  GABAergic synapse  Taste transduction  Neuroactive ligand-receptor interaction |
| 15444 | Hpca | -0.363332961 | down |  |
| 16974 | Lrp6 | -0.365411896 | down | Wnt signaling pathway  Parathyroid hormone synthesis, secretion and action  mTOR signaling pathway |
| 11491 | Adam17 | -0.368686171 | down | Notch signaling pathway |
| 545253 | Gm5820 | -0.376543835 | down |  |
| 77864 | Ypel2 | -0.382104951 | down |  |
| 268977 | Ltbp1 | -0.397295209 | down | TGF-beta signaling pathway |
| 12819 | Col15a1 | -0.399888407 | down | Protein digestion and absorption |
| 15379 | Onecut1 | -0.404519532 | down | Signaling pathways regulating pluripotency of stem cells |
| 319876 | Cobll1 | -0.408191356 | down |  |
| 74199 | Vit | -0.439887785 | down |  |
| 241201 | Cdh7 | -0.442777534 | down |  |
| 320311 | Rnf152 | -0.499168732 | down | mTOR signaling pathway |
| 17387 | Mmp14 | -0.504445135 | down | TNF signaling pathway  Parathyroid hormone synthesis, secretion and action  GnRH signaling pathway |
| 277414 | Trp53i11 | -0.517119423 | down |  |
| 70370 | Fbln7 | -0.52620366 | down |  |
| 14747 | Cmklr1 | -0.548244899 | down |  |
| 246728 | Oas2 | -0.554689343 | down | NOD-like receptor signaling pathway |
| 98845 | Eps8l2 | -0.572319524 | down |  |
| 76960 | Bcas1 | -0.613640797 | down |  |
| 217305 | Cd300ld | -0.651452341 | down |  |
| 235379 | Gldn | -0.66874889 | down |  |
| 232174 | Cyp26b1 | -0.683328444 | down | Metabolic pathways  Retinol metabolism |
| 93842 | Igsf9 | -0.690275483 | down |  |
| 66102 | Cxcl16 | -0.69325817 | down | Chemokine signaling pathway  Cytokine-cytokine receptor interaction |
| 16949 | Loxl1 | -0.822406461 | down |  |
| 13516 | Epyc | -1.038496075 | down |  |
| 242122 | Vtcn1 | -1.192119264 | down | Cell adhesion molecules (CAMs) |
| 13386 | Dlk1 | -1.249631723 | down |  |
| 433182 | Eno1b | -1.600472079 | down | Carbon metabolism  HIF-1 signaling pathway  Metabolic pathways  Biosynthesis of antibiotics  Glycolysis / Gluconeogenesis  Microbial metabolism in diverse environments  Biosynthesis of secondary metabolites  RNA degradation  Biosynthesis of amino acids  Methane metabolism |
